# Evidence of Selective Response in the Free-Breeding Dog Population in Chornobyl

**DOI:** 10.3390/genes17101195

**Published:** 2026-09-27

**Authors:** Megan N. Dillon, Reade B. Roberts, Jennifer A. Betz, Timothy A. Mousseau, Norman J. Kleiman, Matthew Breen

**Affiliations:** 1 Department of Molecular Biomedical Sciences, College of Veterinary Medicine, North Carolina State University, Raleigh, NC 27607, USA; mdillon3@ncsu.edu; 2 Department of Biological Sciences, North Carolina State University, Raleigh, NC 27607, USA; rbrober2@ncsu.edu; 3 Visiting Veterinarians International, Damascus, OR 97089, USA; drbetz@cleanfutures.org (J.A.B.); mousseau@sc.edu (T.A.M.); 4 Clean Futures Fund, Godfrey, IL 62035, USA; 5 Department of Biological Sciences, University of South Carolina, Columbia, SC 29208, USA; 6 Institute for Infectious Disease Translational Research, University of South Carolina, Columbia, SC 29208, USA; 7 Department of Environmental Health Sciences, Mailman School of Public Health, Columbia University, New York, NY 10032, USA; njk3@cumc.columbia.edu; 8 Comparative Medicine Institute, North Carolina State University, Raleigh, NC 27607, USA; 9 Center for Human Health and the Environment, North Carolina State University, Raleigh, NC 27607, USA; 10 Cancer Genetics, UNC Lineberger Comprehensive Cancer Center, University of North Carolina, Chapel Hill, NC 27599, USA; 11 Duke Cancer Institute, Duke University, Durham, NC 27710, USA

**Keywords:** Chornobyl Exclusion Zone, local adaptation, population genetics, genomic resilience, selection, environmental contamination

## Abstract

**Background/Objectives:** Understanding genomic responses to environmental contamination is essential for assessing long-term ecological and genetic risk. The free-breeding dogs inhabiting the Chornobyl Exclusion Zone (CEZ) offer a unique, multigenerational model for investigating these responses within a chronically contaminated landscape. Following the 1986 radioecological disaster at the Chornobyl Nuclear Power Plant, and subsequent remediation activities, the surrounding environment was contaminated by ionizing radiation, heavy metals, organic compounds, and other environmental toxicants. Our previous work identified significant genetic differentiation between two distinct but geographically proximate dog populations within the CEZ, a pattern not explained by breed composition, spatial isolation, or increased mutation rates. **Methods:** In this study, we tested the hypothesis that the dogs residing at the nuclear power plant show genomic signatures consistent with adaptive evolution in response to chronic environmental stressors. This work extends prior population-genetic analyses by integrating whole-genome selection scans with estimates of effective population size and comparative measures of population divergence, providing a framework to evaluate whether the observed genomic differentiation is more consistent with selection than demography. Using these whole genomic data, we evaluated chromosome-level *F_ST_* metrics, estimated effective population size (N_e_), and analyzed a geographically isolated island dog population as a comparative reference for genetic divergence. **Results:** We continued to find that the levels of genetic differentiation are not comparable to other free-breeding dog populations. Genome-wide outlier analyses identified candidate loci showing evidence of positive selection across multiple metrics. **Conclusions:** Genes associated with these loci were enriched for biological functions related to DNA replication and repair, cell death and survival, cellular compromise, immune response, and cancer-associated pathways, suggesting biologically plausible mechanisms through which chronic environmental stress could influence fitness. These findings are consistent with directional selection in the nuclear power plant population, which could potentially be associated with the environmental variables present, and advance our understanding of local adaptation and genomic resilience under chronic environmental contamination.

## 1. Introduction

Genomic responses to anthropogenic environmental change provide important insight into how populations persist, differentiate, and potentially adapt under novel selective pressures. Humans are recognized as the largest ecosystem engineers, and a growing population has increased anthropogenic influence on environments worldwide [1]. Human activities have altered the natural environment through industrialization and urbanization, habitat fragmentation, translocation of non-native species [2,3], and the widespread release of synthetic chemicals and contaminants into ecosystems across the globe [4,5]. Although the intensity and composition of these anthropogenic pressures vary among landscapes, such pollutants are now detectable even in remote environments, including microplastics in the Galápagos Islands and their uptake into local food webs [6]. Understanding whether and how populations respond genetically to such exposures is central to evaluating the evolutionary consequences of persistent environmental contamination.

The Chornobyl Exclusion Zone (CEZ) represents an exceptional system for studying population-genetic consequences of long-term exposure to a heterogeneous contaminated landscape. The 1986 explosion at the Chornobyl Nuclear Power Plant released approximately 5000 petabecquerels of radioisotopes into the surrounding environment, making it the largest nuclear accident in history [7,8,9,10,11]. In addition to radioactive contamination, remediation activities and abandonment of military and industrial infrastructure introduced or left behind heavy metals, organic compounds, pesticides, asbestos, and other environmental toxicants [12,13,14,15]. The persistence of these materials, together with the long half-lives of several radionuclides, has generated chronic exposure risks over the last four decades. Because evacuated towns, villages, and industrial sites remained embedded within this contaminated environment, the CEZ provides a rare opportunity to examine whether free-breeding animal populations show patterns of genomic differentiation consistent with drift, isolation, or selection under sustained environmental stress.

Free-breeding dogs in the CEZ offer a particularly informative model for investigating these questions. During the initial evacuations in 1986, residents were forced to leave companion animals behind, and liquidators were later sent into the CEZ to eradicate these and remaining livestock [14]. It is unclear how many animals were able to evade the liquidators, but enough domestic dogs escaped this fate to establish persistent populations within the zone. Several hundred dogs are now estimated to inhabit the CEZ and are thought to descend, at least in part, from pets left behind after the disaster [14,16]. These populations have likely occupied the contaminated region for more than 30 generations and have become the focus of recent genomic, epidemiological, and pathogen-focused studies [17,18,19,20]. As domestic dogs are effective sentinels for human environmental exposures [21,22], the Chornobyl dog populations provide a valuable system for assessing how chronic exposure may shape genome-wide variation across generations.

Previous studies of Chornobyl dogs identified substantial genetic differentiation between two geographically proximate populations: dogs residing at the nuclear power plant (NPP) and dogs sampled in Chornobyl City (CC), approximately 16 km away [17,18]. The magnitude of differentiation between these populations exceeded estimates among free-breeding dog populations separated by much larger distances across Eastern Europe [17]. Moreover, the CC population was less genetically differentiated from populations hundreds of kilometers away in Eastern Russia, Central Russia, and Poland than from the nearby NPP population [20]. Potential explanations for this pattern, including breed composition, geographic isolation, inbreeding, and increased mutation, have been evaluated but have not definitively accounted for the observed differentiation [17,20]. These findings raise the possibility that non-neutral evolutionary processes may have contributed to the distinctive genomic profile of the NPP population.

Here, we extend our prior population-genetic analyses by evaluating whether the genetic differentiation of the NPP population is more consistent with random genetic drift or directional selection. Genetic drift, potentially amplified by founder effects, bottlenecks, limited recent admixture, or reduced effective population size, could contribute to divergence between the NPP and CC populations. However, preliminary selection scans using SNP-array data identified putative outlier loci in the NPP population, suggesting that selective processes may also be involved [17]. Given the persistence of the NPP dogs in a chronically contaminated environment for more than 30 generations, sustained exposure to ionizing radiation, heavy metals, organic compounds, and other toxicants may have imposed selective pressures detectable in genome-wide patterns of differentiation. In this study, we integrated whole-genome sequence data with estimates of effective population size, comparisons to isolated island dog populations, chromosome-level *F_ST_* analyses, genome-wide outlier detection, extended haplotype homozygosity, nucleotide diversity, and Tajima’s D to test the hypothesis that the NPP population bears genomic signatures consistent with directional selection. As many *F_ST_*-outlier and haplotype sharing methods for detecting signatures of directional selection can generate false-positives brought on by other demographic changes, we utilize a more robust method for detecting *F_ST_*-outliers and couple this with other metrics to attempt to alleviate some of these non-target trends. By distinguishing selection-associated signals from patterns expected under drift and isolation, this work advances understanding of local adaptation and genomic resilience in a free-breeding mammalian population exposed to chronic environmental contamination.

## 2. Materials and Methods

### 2.1. Initial Filtering for WGS Data

Previously, courtesy of NHGRI, we acquired whole genome sequence (WGS) data for 106 individuals, sampled in 2018 and 2019, from the Nuclear Power Plant (NPP, *n* = 58) and Chornobyl City (CC, *n* = 48), from which we called and processed SNPs (detailed further in Dillon et al. [20]). This dataset contained autosomal genotypes for 13,532,206 SNPs. To prepare for the analyses described in this work, we further filtered these data and removed individuals with first degree relationships with plink v2.00a6 M1 (--king-cutoff-table 0.177) along with those individuals that, through a discriminant analysis of principal component cluster (DAPC; described in Dillon et al. [17]), group with the comparison population [23]. This allowed us to assess individuals that best captured the allelic frequencies of each population as we aimed to better understand evolutionary histories for these two independent populations. These data, along with SNP genotypes of 712,000 loci for 60 NPP and 56 CC individuals [17], were then used for one of the analyses described below. All individuals with WGS data are also represented in the SNP dataset.

### 2.2. Effective Population Size Estimates

To address the possibility of genetic drift driving the observed genetic differentiation, we first estimated the effective population sizes (Ne) for both the NPP and CC populations to determine whether the former was substantially lower. Small population sizes can accelerate the effects of genetic drift. We used NeEstimator v2.1 to estimate the Ne for both the NPP and CC groups with the WGS data [24]. First, we assessed Ne across the genome after filtering loci and randomly selecting 5000 SNPs (plink2 --geno 0, --maf 0.05, --thin-count 5000). We then generated a genepop input file with the dartR package in R (v. 2.9.9.5) [25]. With NeEstimator v2, we used default settings and assessed Ne with all three methods: molecular co-ancestry [26]; linkage disequilibrium (LD) [27,28]; and heterozygote excess [29,30] for critical, or minimum, allele frequencies of 0.05, 0.02, and 0.01. We repeated this three times with different randomly selected sets of 5000 loci. To ensure continuity over a denser dataset, we repeated the NeEstimator analysis on a set of 21,955 SNP loci separated by 100 kb (plink2 --geno 0, --maf 0.05, --bp-space 100000). These data did not undergo stringent LD pruning, as one of the methods of estimation considers LD.

We next assessed Ne at the chromosome level. It has previously been asserted that Ne can fluctuate across the genome as influenced by selection and mutation, but the typical expectation for drift, however, is that neutral sites across the genome will be affected at similar rates [31]. For this analysis, we first filtered the dataset for quality with plink (plink2 --geno 0.50, --mac 2, --snps-only) and selected SNPs that were separated by at least 7500 bp (plink2 --bp-space 7500). This, in contrast to a set number of loci across all autosomes, produced datasets that were proportional to the length of each autosome, ranging from 15,441 (chromosome 1) to 3095 loci (chromosome 38) and allowed for the removal of more closely linked loci. This filter metric was applied at this distance so as not to remove more historical LD trends, which are informative for assessing Ne in this method. We conducted each chromosome-level analysis in NeEstimator, as described previously for the whole genome assessments.

In conjunction with contemporary estimations of Ne, we also assessed historical trends in Ne across the last 100 generations in order to detect possible bottlenecks. We conducted this analysis with GONE, a linkage disequilibrium (LD)-based algorithm for assessing historical Ne [32]. We performed GONE analysis on a dataset of 500,000 randomly selected SNP loci across the genome with missingness below 90% (plink2 --thin-count 500000, --geno 0.9, random number seed: 1737501509) and used a generalized recombination rate of 0.97 cM per megabase [33]. This missingness threshold is notably not conservative for this particular dataset, but this allowed for the retention of potentially rare alleles. Less than 0.5% of loci included had missing data for over 50% of individuals.

### 2.3. Island Population Evaluation

To evaluate whether the previously identified levels of genetic differentiation for the NPP population mimicked that of an isolated population, we repeated some of our previous population genetic analyses with the inclusion of isolated, island populations: dogs from the Galápagos islands. The dogs in the Galapagos archipelago have a similar history of isolation, little recent admixture, and an attempted culling some 30 years ago. They serve, therefore, as an effective comparison group with comparable historical isolation but without the environmental contamination found in the Chornobyl region. Genotypes for these dogs from the CanineHD Whole-Genome Genotyping BeadChip array (Illumina, Inc., San Diego, CA, USA) were acquired from Spatola et al. [34] and converted into plink format. These data were then merged with a genetic dataset containing SNP genotypes for the NPP and CC populations Dillon et al. (2024) [20], as well as Eastern European village dogs from Poland, Eastern Russia, and Central Russia from Pilot et al. [35]. The combined data were filtered to remove individuals with first-degree relationships (plink2 --king-cutoff-table 0.177), and loci were filtered to remove SNPs with more than 50% missing data and with a minor allele frequency lower than 5% (plink2 --geno 0.5, --maf 0.05). We calculated pairwise *F_ST_* across the populations (plink2 --fst --within) and then conducted a Discriminant Analysis of Principal Components (DAPC) with adegenet (v. 2.1.11) [36]. The find.clusters function was used to identify the number of clusters with the lowest Bayesian Information Criterion (BIC) that best fit the data, and a cross-validation procedure was used to identify the optimum number of principal components to retain in the DAPC. After running the analysis for the inferred clusters (adegenet: dapc), the DAPC was visualized.

### 2.4. F_ST_ Estimates per Chromosome

We next assessed whether the level of genetic differentiation between the NPP population and other dog populations was consistent across the genome or specific to certain genomic regions by calculating *F_ST_* per chromosome. Since genetic drift is a neutral, stochastic process, we hypothesized that these estimates should be similar across all chromosomes if genetic drift is the predominant force influencing the differentiation. We calculated these with both the WGS data and the reduced density SNP dataset containing free-breeding dog populations. For both estimates, we incorporated additional comparison groups to better contextualize deviations across the chromosomes and to decipher whether any trends were consistent across comparisons.

Firstly, to assess the *F_ST_* across the WGS data, we combined our genotype data with that from Morrill et al. [37] to generate a dataset that contained SNP genotypes for four different populations. These populations included the NPP (*n* = 33), CC (*n* = 30), and mixed-breed dogs (*n* = 22), and an additional group comprising 24 purebred dogs. For this purebred ‘population’, we included three dogs each from eight breeds selected to represent a distinct clade based on the breed cladogram in Parker et al. [38]. The eight breeds used were bearded collie, Belgian Tervuren, Bernese mountain dog, bull terrier, Chihuahua, Jack Russell terrier, German shepherd dog, and golden retriever. This fourth group was selected to represent broad genetic ancestry and to likely contain sufficient diversity at each locus so as to prevent genomic outliers based on breed-specific genotypes and past artificial selection; when considering these individuals collectively at the population level, we posit that they would more closely resemble the other admixed populations that were included. To ensure this was the case, we performed a principal components analysis (PCA) in adegenet (glPca) with a randomly selected set of 500,000 SNP loci before moving forward (plink2 --thin-count; random number seed: 1730316660).

We then calculated pairwise *F_ST_* across all six combinations of pairs with the whole genome sequence data. This file was filtered to remove loci with low minor allele frequencies and missing data (plink2 --geno 0, --maf 0.05). We first estimated *F_ST_* across all 4,828,448 filtered loci, and then for each chromosome (plink2 --fst –within; --chr N). We next calculated the change for each chromosome estimate when compared to the overall estimate considering all autosomal loci (hereafter, all-autosomal) for that pair. To better assess how the deviation in *F_ST_* across each chromosome compared to other free-breeding dog populations, we repeated these calculations for the reduced density SNP dataset containing the NPP, CC, Polish, Eastern Russian, Central Russian, and Galápagos dog populations.

### 2.5. OutFLANK Outlier Detection

To build on our previous scans for evidence of directional selection, we conducted an outlier analysis with the WGS data with OutFLANK [39]. This method has a lower false positivity rate for calling outliers than similar programs and is compatible with WGS data. Due to the algorithm detecting outliers based on higher-than-expected *F_ST_* measures based on the locus’s expected heterozygosity, it provides more meaningful results when considering more populations. Therefore, we used the dataset described previously for *F_ST_* calculations, which consisted of the NPP population, CC population, mixed-breed dogs, and the group made up of 24 purebred dogs.

We performed the outlier analysis using OutFLANK (v 0.2) in R, after selecting only those loci that had no missing genotypes and a minimum minor allele frequency of 5% (plink2 --geno 0, --maf 0.05). To accommodate the high number of considered loci, we split the chromosomes into three separate groups and considered chromosomes 1–10, 11–20, and 21–38 separately for the initial preparations. For each set of chromosomes, we prepared the *F_ST_* matrix (MakeDiploidFSTMat) and then fit a curve to these *F_ST_* data (OutFLANK; Chromosome 1–10 Trim Fractions: 0.1, 0.3; Chromosome 11–20 Trim Fractions: 0.1, 0.35; Chromosome 21–38 Trim Fractions: 0.1, 0.3). We adjusted the trim settings for both the right and left of this *F_ST_* distribution to fit a normal curve that best represented the neutral *F_ST_* distribution. We used a q-value threshold of 0.1 to identify potential outliers which allowed for a higher false discovery rate, since we used this data in conjunction with additional analyses and also sought to accommodate the lack of fully differentiated loci within the NPP population.

For each resultant outlier locus, we assessed the locus *F_ST_* estimates and allele frequencies across each population to determine which population was the most differentiated and also to determine whether the allele frequencies were consistent for loci in close proximity to each other. With this, we determined which loci were outliers specifically for the NPP population and whether any genomic regions had multiple outlier loci with trends resembling selective sweeps (i.e., multiple loci close in proximity with consistent allele frequencies). After identifying the loci that were putative outliers for the NPP population, we identified genes within 50 kb of each of the genomic regions or single loci with the University of California Santa Cruz (UCSC) genome browser for CanFam3 [40]. The genes identified within these outlier regions were then analyzed through Ingenuity Pathway Analysis (IPA; QIAGEN Inc., Venlo, Netherlands) through the expression analysis [41].

### 2.6. Cross Population-Extended Haplotype Homozygosity (XP-EHH)

To better assess evidence of selection, we searched for evidence of selected sweeps through cross population extended haplotype homozygosity (XP-EHH). Before conducting the analysis, we used ShapeIt2 for statistical phasing of the WGS data for the NPP and CC populations [42], along with the purebred and mixed-breed groups from the OutFLANK analysis. The ShapeIt2 input files contained just one chromosome each and the SNP data were pruned to remove loci with missing allele frequencies above 10% (plink2 --geno 0.1). ShapeIt2 was run with a window setting of 0.5 and used the genetic map from Campbell et al. [43] for recombination rates. These data files were then split into each population, filtered with plink2 (--maf 0.05, --geno 0), and processed with the REHH package in R [44]. A scan was performed across each autosome to calculate integrated haplotype homozygosity scores and compared between the NPP to each of the comparison populations for XP-EHH calculations. We then assessed the XP-EHH scores for each of the outlier regions from the OutFLANK analysis to better identify evidence of directional selection. We ordered the calculation of ratios across the comparisons so that positive scores for XP-EHH would indicate evidence of selection in the NPP population (i.e., NPP vs. CC, NPP vs. Mixed, NPP vs. Purebred).

### 2.7. Sliding Window F_ST_, Nucleotide Diversity, and Tajima’s D

As an additional measure for evidence of selection and to better assess the genetic trends within the outlier regions, we calculated sliding window *F_ST_*, sliding window nucleotide diversity (π), and window-based Tajima’s D across the whole genome data. Nucleotide diversity can be decreased by directional selection in certain stretches of the genome and across the genome by genetic drift. The fixation index, *F_ST_*, is increased in regions of differentiation between populations. We calculated nucleotide diversity and pairwise *F_ST_* for all of the populations studied in the OutFLANK analysis across windows of 5 kb and 10 kb with step sizes of 500 bp and 1000 bp, respectively. These measures were calculated in vcftools v 0.1.17 with the window-pi and fst-window-size commands [45]. Tajima’s D compares two measures of genetic diversity to assess neutral and non-neutral evolutionary signatures [46]. A negative value of Tajima’s D suggests that the considered DNA segments have violated neutrality and may have experienced directional selection or a recent population expansion. We calculated Tajima’s D over 5 kb and 10 kb windows using vcftools v 0.1.17 (--TajimaD 5000; --TajimaD 10000) for all of the populations. We then assessed these estimations across chromosomes with identified outlier loci by visualizing these data across each chromosome for all populations. After assessing the calculations for the outlier regions, we conducted an additional IPA analysis on the genes that were present in the regions that with additional evidence suggestive of selection for at least three of the four subsequently calculated metrics, including XP-EHH, sliding window *F_ST_*, pi, and Tajima’s D.

## 3. Results

### 3.1. Initial Filtering for WGS Data

Of the initial WGS data for 106 individuals (NPP, *n* = 58; CC, *n* = 48), we removed 34 related individuals (NPP, *n* = 20; CC, *n* = 14) and 9 individuals that clustered with the opposite populations (NPP, *n* = 5; CC, *n* = 4). This left 63 individuals for subsequent analyses that were the most location specific, 33 from NPP and 30 from CC. The data for these individuals were analyzed as follows.

### 3.2. Effective Population Size Estimates

Overall Ne estimates did not highlight a consistent significant difference for the effective number of breeding individuals between CC and the NPP based on molecular co-ancestry or heterozygosity excess, when assessed across all autosomes or when assessed per chromosome (Table 1 and Table S1). The linkage disequilibrium method, however, did highlight a substantially lower Ne for the Nuclear Power Plant when assessed at the chromosome level across loci separated by 7.5 kb (Table S1). Over all autosomes, the linkage disequilibrium estimates were not significantly different as per the jackknife confidence intervals, but the CC population had slightly higher estimates.

The assessment for historical shifts in population size through GONE indicated a decline in the NPP population size starting some 35–40 generations ago, which is consistent with the timing of the 1986 nuclear disaster and subsequent culling by liquidators (Figure 1). This trend was not evident in the CC population, but we did note a lower ancestral population size in the CC population compared to the NPP population. The GONE analysis did indicate, however, a decline in the CC population beginning eight generations prior to 2019.

### 3.3. Island Population Evaluation

After filtering the combined dataset for quality and to remove related individuals, 100,413 SNP loci, and data for 250 dogs were retained for analysis: NPP, *n* = 33; CC, *n* = 32; Poland, *n* = 18; Eastern Russia, *n* = 15; Central Russia, *n* = 13; Isabela Island, *n* = 26; Santa Cruz Island, *n* = 97; Galápagos Hunting Dogs, *n* = 16. Pairwise *F_ST_* estimates between the populations highlight high degrees of genetic differentiation between the NPP and all other populations, ranging from 0.039 (NPP and CC) to 0.077 (Isabela Island and NPP; Table 2). All other population comparisons range between 0.006 and (Eastern Russia and Central Russia; Poland) to 0.039 (CC and Isabela Island).

The discriminant analysis of principal components (DAPC) recapitulated the findings of the *F_ST_* assessment, highlighting a higher degree of variance between the NPP and all other populations than that found between the Galápagos Islands and European populations. Genetic differences among the eight considered populations were captured in just three clusters, with cluster 1 containing only NPP individuals (Figure 2). Discriminant axis one (eigenvalue = 1644.6) explains more than twice the amount of variance as axis two (eigenvalue = 658.4), and axis one serves primarily to differentiate the NPP cluster from all others (Figure 2C).

### 3.4. F_ST_ Estimates per Chromosome

The preliminary PCA for the four considered groups indicated fairly distinct groupings for NPP, CC and the mixed-breed groups (Figure S1). The purebred group had higher amounts of genetic diversity but still clustered closely to the mixed-breed group and CC population. After verifying that the purebred group resembled an admixed population instead of serving as a distant outlier, we included this group in the *F_ST_* analysis.

The fixation index highlighted the NPP population as the most differentiated group with all loci considered for the all-autosomal estimates, with *F_ST_* ranging from 0.052 (versus CC) to 0.068 (versus purebred), while the mixed-breed and purebred groups had the lowest levels of genetic differentiation (*F_ST_* = 0.013; Table 3). In addition to the higher degree of differentiation, we found more variability across the individual autosomal *F_ST_* estimates when the NPP population was included, and this same level of variation was not found in the other comparisons (Figure 3A). Pairwise *F_ST_* for each chromosome with the NPP population (including comparisons with CC, mixed-breed, and purebred groups) deviated from the all-autosomal estimates by 0.008 on average, while other pairwise estimates varied only by an average of 0.002 (Figure 3C). For the NPP population, an *F_ST_* estimate for a single chromosome varied as much as 0.032 and −0.024 from the all-autosomal estimate, while the maximum change for other comparisons was 0.009. The chromosomes with the most variation in genetic differentiation were consistently high or low across all three considered comparisons; for example, chromosome 8 was more differentiated for the NPP than other chromosomes when compared to the CC population (*F_ST_* = 0.081), mixed-breed group (*F_ST_* = 0.089), and the purebred group (*F_ST_* = 0.10). In contrast, chromosome 8 did not show consistent changes from the all-autosomal estimates for CC (versus mixed breed, change in *F_ST_* = −0.0016; versus breed, change in *F_ST_* = 0.00038).

The observed trends in variability across the chromosomes is supported in the reduced density SNP dataset with the additional free-breeding populations included (Figure S2). Pairwise *F_ST_* estimates which include the NPP population are considerably more variable across the chromosomes, with estimates varying from the whole genome estimate on average by 0.0074, than those which do not include the NPP, which deviate by 0.0035 on average. Across the analysis of both datasets, chromosomes 8, 26, and 27 are the most differentiated chromosomes for the NPP population, with consistently high *F_ST_* estimations and the largest deviations from the whole genome estimates.

### 3.5. OutFLANK Outlier Detection

The OutFLANK analysis identified 1574 outlier loci from the 4,828,448 SNP loci in the input files. Of these, 1138 loci were outliers for the NPP population when the allele frequencies of each population were analyzed (Figure 4A,B; Table S2). The identified outlier loci were distributed across 29 of the 38 autosomes. Some chromosomes, such as chromosome 27, contain multiple outlier loci across different regions with variable *F_ST_* and expected heterozygosity (e.g., Figure 4C,D; see Figure S3).

Eighty-six genomic regions were identified that contained multiple significant outlier loci. A total of 1094 outlier loci were in close proximity to another outlier locus and were separated on average by 7 kb. Only 44 of the identified outlier loci were isolated in the genome by distances greater than 100 kb. Examination of the 130 genomic regions surrounding all 1138 outlier loci identified 247 genes within 50 kb of an outlier loci or within the identified regions (Table S2). IPA highlighted cancer, including urogenital cancer and melanoma, as the top disease associated with these genes, with *p*-values ranging from 1.89 × 10^−2^ to 7.44 × 10^−27^ (Figure S4). Three of the top molecular and cellular functions were “DNA Replication, Recombination, and Repair”, “Cell Death and Survival”, and “Cellular Compromise”. IPA identified 17 networks within the gene input, and the top diseases and functions for these networks included the same three molecular and cellular functions and also highlighted “Cancer”, “Cellular Function and Maintenance”, “Inflammatory Response”, “Infectious Diseases”, and “Cell Cycle” (Table 4). Many of the identified genes are also associated with other immune functions, including immune cell trafficking, humoral immune response, and antimicrobial response. Additionally, one outlier locus on chromosome 8 is located in an intronic region of the T-cell receptor alpha chain C region.

### 3.6. Cross Population-Extended Haplotype Homozygosity (XP-EHH)

The calculations of XP-EHH for each chromosome and each pair tended to recapitulate the findings of the OutFLANK analysis. We found that most of the outlier loci within the defined outlier regions corresponded with high, positive XP-EHH values (e.g., Figure 4E; Table S2). Of the outlier regions and independent loci identified with OutFLANK, 111 (89.5%) of these 124 regions also have positive ratios of XP-EHH across the three comparisons, and these values often peaked within these genomic regions (Figure S3). Notably, the regions that contain multiple outlier loci frequently had higher XP-EHH, and these locations also share more consistencies across the three different pairwise comparisons to the NPP population.

### 3.7. Sliding Window F_ST_, Nucleotide Diversity, and Tajima’s D

The calculation of additional metrics continued to reinforce many of the OutFLANK outlier regions (e.g., Figure 4F–H and Figure S3). Much like with the XP-EHH calculations, the regions containing multiple outliers also displayed consistency in *F_ST_*, nucleotide diversity, and Tajima’s D. Sliding window *F_ST_* analysis closely resembled the *F_ST_* calculated through OutFLANK for each locus, but with a higher degree of detail and added separation between different pairwise comparisons (i.e., NPP versus CC compared to NPP versus the Mix group). For both nucleotide diversity and Tajima’s D plots at the chromosome level, there are correlations between local minima and the OutFLANK results for the largest outlier regions, as is apparent in the middle region of chromosome 27 (see Figure 4F,H). The smaller regions or regions with only one outlier locus, however, are not as well explained by the whole chromosome view which smoothed out the noise. These required additional assessment of the individual regions across a smaller genomic range, upon which we noted negative Tajima’s D values and decreased nucleotide diversity for 75 and 108 of these regions, respectively (Table S2). A total of 111 regions were supported by the results of at least three of these four analyses that were used to follow up on the OutFLANK results, and all of the regions were supported by at least two of the additional metrics. The second IPA conducted for these outlier regions with the highest confidence upheld the findings from the previous analysis, with cancer as the top disease (*p*-values ranging from 2.38 × 10^−2^ to 1.65 × 10^−24^). “DNA Replication, Recombination, and Repair”, “Cell Death and Survival”, and “Cellular Compromise” were maintained among the top five molecular and cellular functions.

## 4. Discussion

Although extensive remediation efforts followed the 1986 accident, many areas surrounding the Chornobyl Nuclear Power Plant remain contaminated by radioactivity and a complex mixture of additional environmental hazards, including heavy metals and organic compounds. Despite these conditions, a wide range of plant, insect, and animal life continue to survive and, in some cases, even thrive in some of the most contaminated areas. Before the 2022 Russian invasion, numerous human workers, residents, and military personnel entered or temporarily resided within the CEZ, particularly in Chornobyl City. The persistence of large numbers of free-breeding dogs within the CEZ provides a unique opportunity to evaluate multigenerational genomic responses to chronic environmental exposure and relate such findings to potential human health risks. In this study, we extended our previous work on Chornobyl dog population structure by evaluating whether the marked genetic differentiation between two geographically close populations is more consistent with genetic drift or directional selection. Overall, our findings suggest that genetic drift is unlikely to entirely explain the observed genetic divergence and instead supports a role for directional selection in shaping the genomic profile of the NPP population.

Our results are consistent with prior published work that the NPP population is genetically differentiated from other free-breeding dog populations [17,20]. However, the pattern of genetic variation in the NPP population differed from other geographically isolated dog populations. Compared with free-breeding populations in the Galápagos Islands, the NPP population was highly differentiated from all comparison populations including the CC population, just 16 km away. Remarkably, Galápagos dog populations were more genetically similar to dog populations in CC and across Europe than to the NPP population. Chromosome-level *F_ST_* analyses further showed that comparisons involving the NPP population exhibited greater variability across autosomes than comparisons among other population pairs. Although genetic drift can generate genome-wide divergence, neutral loci are expected to be affected across the genome in a similar manner under an assumed constant mutation rate [47]. In marked contrast, our findings indicate that several chromosomes, particularly 8, 14, 26, and 27, were consistently more differentiated in comparisons involving the NPP population. The NPP population also did not show evidence of reduced genome-wide nucleotide diversity relative to the other groups analyzed. Taken together, the contrast with island populations, maintenance of nucleotide diversity, and elevation of *F_ST_* suggests that genetic drift alone is insufficient to explain the distinctive genomic differentiation of the NPP population. Instead, the high fluctuation in differentiation between chromosomes, similar rates of nucleotide diversity, and the stark contrast in genetic variation for the NPP population compared to that of the island dog populations points towards an additional, non-drift driver of the genetic differentiation.

Effective population size estimates provided additional evidence that population size differences alone do not account for the observed differentiation. If the NPP population had experienced a uniquely severe reduction in effective population size, isolation and/or drift would be more plausible explanations for the observed differences. However, we found that the effective population size and effective number of breeder estimates of the NPP and CC populations were not significantly different by heterozygote excess, molecular coancestry, or overall estimates of linkage disequilibrium. At the chromosome level, we did note a reduction in effective population size when assessed via linkage disequilibrium, but these analyses used loci separated by only 7.5 kb. As selection can influence local linkage disequilibrium, these chromosomal-level estimates may themselves be affected by non-neutral processes or may simply indicate some non-significant differences in these population estimates [48]. In all, though the NPP and CC population sizes are relatively small by some measures, the lack of significant differences between the two populations argues against a reduced population size alone as the primary driver of the NPP-specific differentiation.

In estimating historical trends in effective population sizes, we potentially identified evidence for a population decline in the NPP population occurring some 35 generations ago, broadly consistent with the timing of the 1986 disaster and subsequent culling efforts. This decline could reflect a bottleneck, early mortality associated with acute environmental exposure, or selection acting on the genetic variation at that time. If there was indeed strong selection on the NPP population, a decline would be expected as there would be variable fitness across genotypes. The heightened historical population size for the NPP that predates the decline may be an artifact of the high genetic diversity and lack of relationships that were present at the time of the disaster. It is likely that the current dog populations are mostly descendants of household pets from Pripyat, the city abandoned 36 h after the disaster that housed workers and their families, which is approximately 3 km from the NPP. This hypothesis would suggest that the founding populations were assembled with predominantly unrelated, diverse individuals. These trends were not replicated for the CC population; however, the historical population analysis with GONE did suggest a minor bottleneck occurring in the CC population around nine generations ago. This does not align with the 1986 accident or the commencement of the sterilization program in 2016, and so cannot be attributed to a specific known event. Potential contributors include recent disease outbreaks, culling of stray dogs around Kyiv ahead of the Euro 2012 football championship, local inbreeding events, or low levels of migration between the two populations (suggested in Dillon et al. [17]), which may bias the result when population sizes differ [49]. These results support the view that demographic history may have contributed to population structure but does not fully explain the genomic patterns observed in the NPP population.

Consistent with our previous preliminary scans [17], the current WGS-based analyses identified evidence of directional selection at over 100 candidate loci in the NPP population. Many of these loci are supported by complementary metrics, including Tajima’s D and XP-EHH. When compared to the additional data generated within this study, such as the per chromosome *F_ST_* estimates, some corresponding trends were noted. Chromosomes with no identified outlier loci tended to have more extreme decreases in *F_ST_*, as can be seen with chromosomes 6, 16, 20, 24, and 29. The identified outliers, however, were often located on the chromosomes with the highest *F_ST_* estimates, including chromosomes 8, 14, 26, and 27, highlighting the expanse of the differentiation in the outlier regions. Importantly, every outlier region or individual locus was supported by at least two follow-up analyses, and 111 regions, comprising 1093 outlier loci were supported by at least three analytical approaches (i.e., OutFLANK, XP-EHH, and sliding window *F_ST_*). The concordance across independent selection metrics strengthens the interpretation that these candidate loci contribute to NPP-specific differentiation.

Interestingly, the genes located around these outlier loci are associated with cancer development, DNA replication and repair, cell cycle functions, and immune function. Further work is required to validate these outliers, but these data suggest that some directional selective pressures were at play related to these diseases and pathways. Møller and Mousseau [50] reviewed a number of studies that looked for evidence of local adaption in populations within the Chornobyl Exclusion Zone, identifying some trends that span across taxonomic groups. Specifically, some of these studies highlight oxidative stress adaptation in birds [51] and improved DNA repair capacity in silver birch and evening primrose [52]. A more recent study reported expression analyses of grey wolves within the Exclusion Zone that provided evidence that suggested selective adaptation in a smaller subset of samples, and some of the differentially expressed genes in this population are associated with DNA damage and immunological pathways [53]. In comparison to Love et al. [53], our study reports findings at the genomic level. Additional gene expression studies of the Chornobyl dog populations may add support to the possibility of selection of pathways which may have protected the dogs’ genome from potentially mutagenic compounds. One plausible scenario is that after the disaster, existing population genetic variation may have provided protection to some dogs from the development of adolescent cancers (i.e., cancers that develop pre-sexual maturity), heightened immunological surveillance, or the early lethal effects of various toxic exposures that prompted strong selective responses. Inflammatory processes also appear as an associated function in one of the top gene networks, and studies suggest that the inflammatory response is triggered by exposure to ionizing radiation (reviewed in Mukherjee et al. [54]), as well as other toxic exposures. A reduction in the inflammatory response may have provided a fitness advantage and prevented the accumulation of mutations or resistance to disease in the early generations of these dogs.

Overall, this manuscript provides strong evidence that the high level of genetic differentiation between the NPP population and other populations of free-breeding dogs is not solely due to random genetic drift. We did not find evidence of similarly high levels of genetic differentiation for island populations that have faced a similar history of isolation. These data do indicate that genetic differentiation is highly concentrated to specific chromosomal regions. This work expands upon our previous selection scans by including additional population comparisons and deeper assessment of other genetic measures, such as XP-EHH and Tajima’s D, to provide stronger evidence for selective pressures and specific loci. The findings provide strong support for directional selection playing an important role in shaping the evolutionary history of the NPP dog population.

## 5. Conclusions

This paper provides evidence for directional selection within a population of free-breeding dogs that are thought to have occupied the contaminated region immediately surrounding the Chornobyl Nuclear Power Plant for over 40 years. Though it is difficult to chart the specifics of their evolutionary history without historical biospecimens from the dogs and ecological samples of the diversity of contaminants present, it is likely that the Chornobyl Nuclear Power Plant dogs have adapted over the past 35 or so generations, and it may be tied to their unique environmental circumstances. The extant literature is clear that domestic dogs, with their rich evolutionary history and large degree of underlying genetic diversity, are an excellent model for assessing selection effects. The findings from this manuscript add to and extend those findings using a unique and valuable canine resource. Continued analysis of Chornobyl dog populations is critical to confirm our findings and additional study will likely provide additional critical insights into the adverse health effects and genetic response to multi-generational exposure to ionizing radiation, heavy metals, organics, and other environmental toxicants. As nuclear power generation expands worldwide to meet growing energy demands, most notably recently from AI computational demands [55,56], it is likely that additional nuclear power accidents will occur, with similar ecological and environmental contamination concerns as those posed by the Chornobyl and Fukushima disasters. Furthermore, anthropogenic contamination is increasing worldwide as nanoplastics, accelerating industrial activities, and thousands of new man-made chemicals and materials enter our environment and the waste stream. It is not an understatement to conclude that the human health risks posed by future environmental and ecological disasters require continued investigation into the genetic and physiological bases of human susceptibility, adaptation, and resilience to environmental hazards.

## Figures and Tables

**Figure 1:**
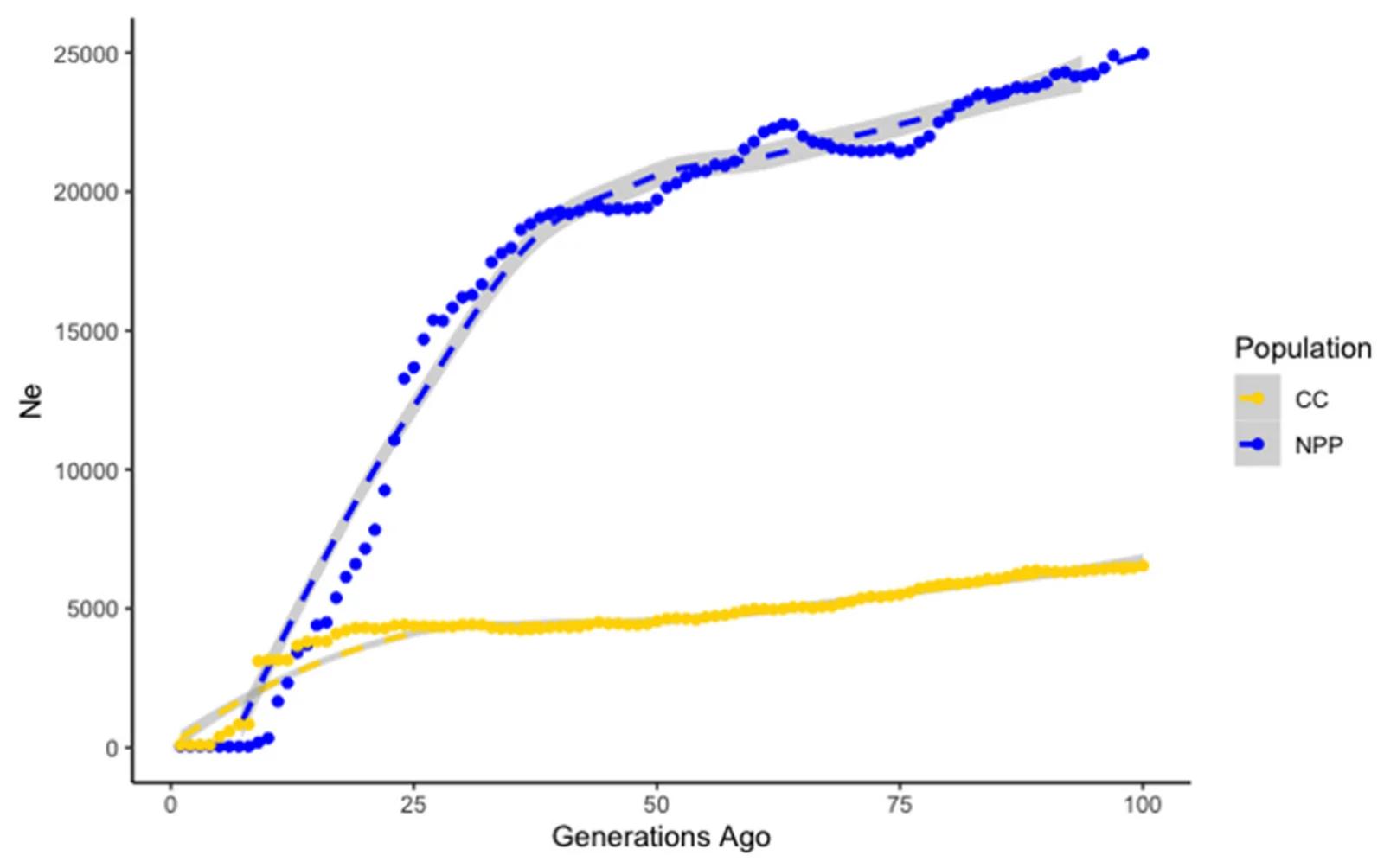
Historical effective population size (Ne) estimations generated through GONE analysis. The plot visualizes the Ne as estimated for the past 100 generations for each population, with points for the population size estimates and a loess smoother for these data points with default confidence band in gray. CC indicates Chornobyl City (in gold) and NPP indicates the Nuclear Power Plant population (in blue).

**Figure 2:**
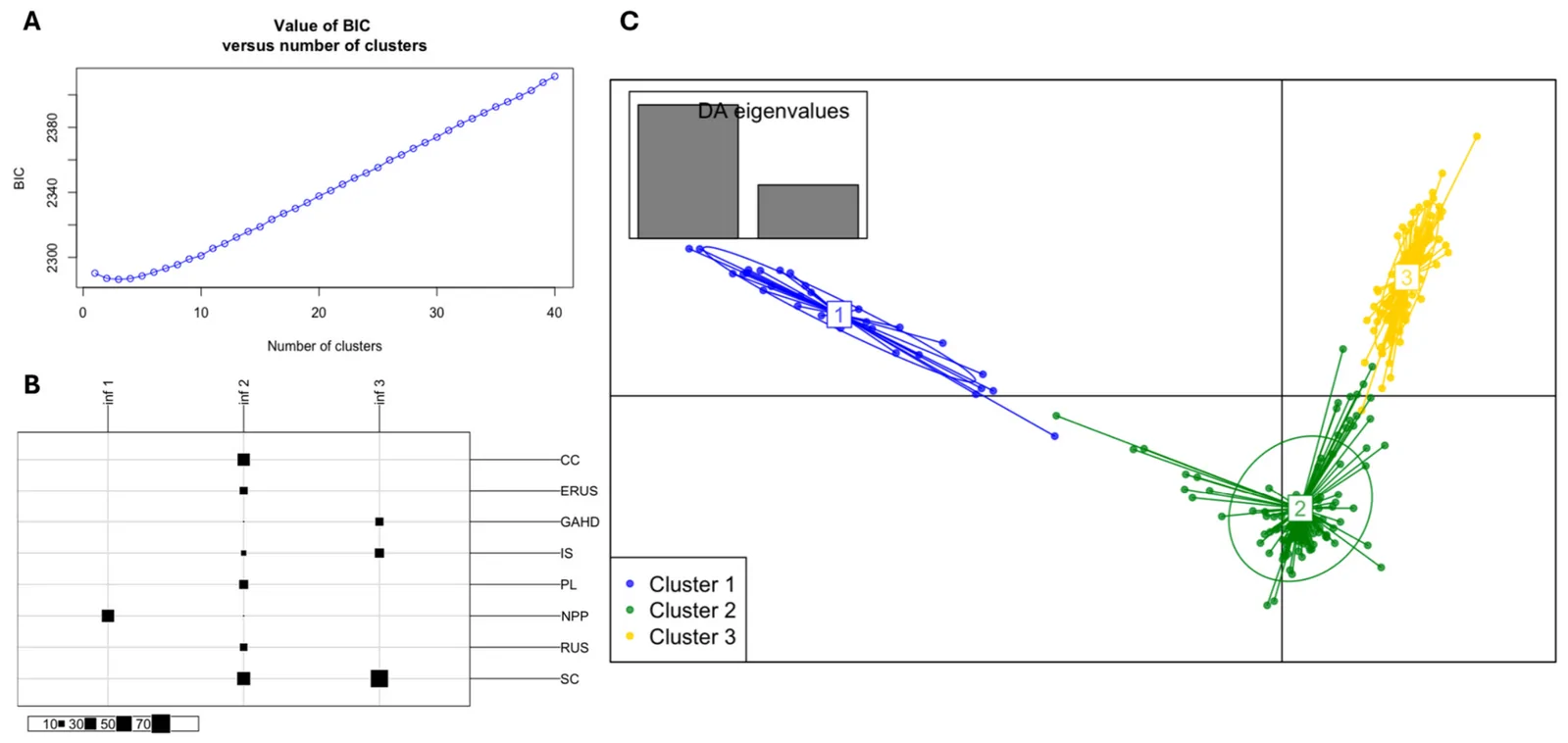
Discriminant Analysis of Principal Components (DAPC) results with the reduced density SNP dataset for eight free-breeding dog populations. This cluster analysis was performed based on optimum Bayesian Information Criterion (BIC) values, which indicated that three clusters best group the data (**A**). The three inferred clusters (inf 1–3), with resultant population assignment indicated by size of the square (**B**), were then used for the DAPC (**C**). DAPC was run with two discriminant axes and 20 principal components. Inf 1–3 indicates the inferred cluster makeup based on the input population. CC is Chornobyl City, ERUS is Eastern Russia, GAHD is Galapagos Hunting Dogs, IS is Isabela Island, PL is Poland, NPP is Nuclear Power Plant, RUS is Russia, and SC is Santa Cruz Island.

**Figure 3:**
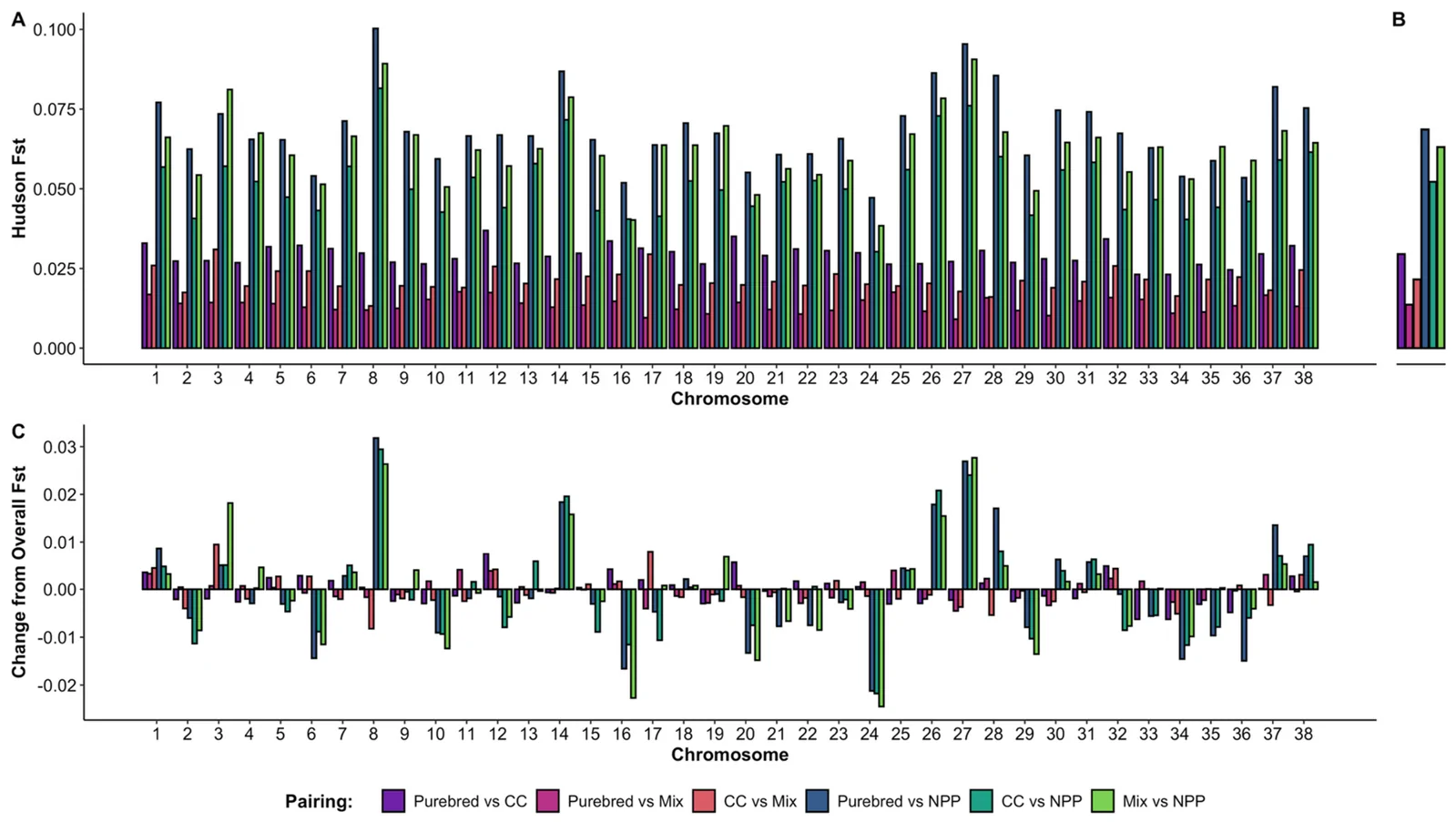
Pairwise *F_ST_* estimates across each autosome and the amount of deviation from the all-autosomal estimates for groups within the whole genome sequence dataset. Hudson *F_ST_* was estimated for each pairing in the WGS dataset for each autosome, color coded for each pairwise assessment (**A**) and across all autosomal loci (**B**). The difference between each chromosomal estimate and the all-autosomal estimate is also plotted for each pair as the change from overall *F_ST_* (**C**). Comparisons involving the NPP population are color-coded in shades of blue and green. NPP is Nuclear Power Plant and CC is Chornobyl City.

**Figure 4:**
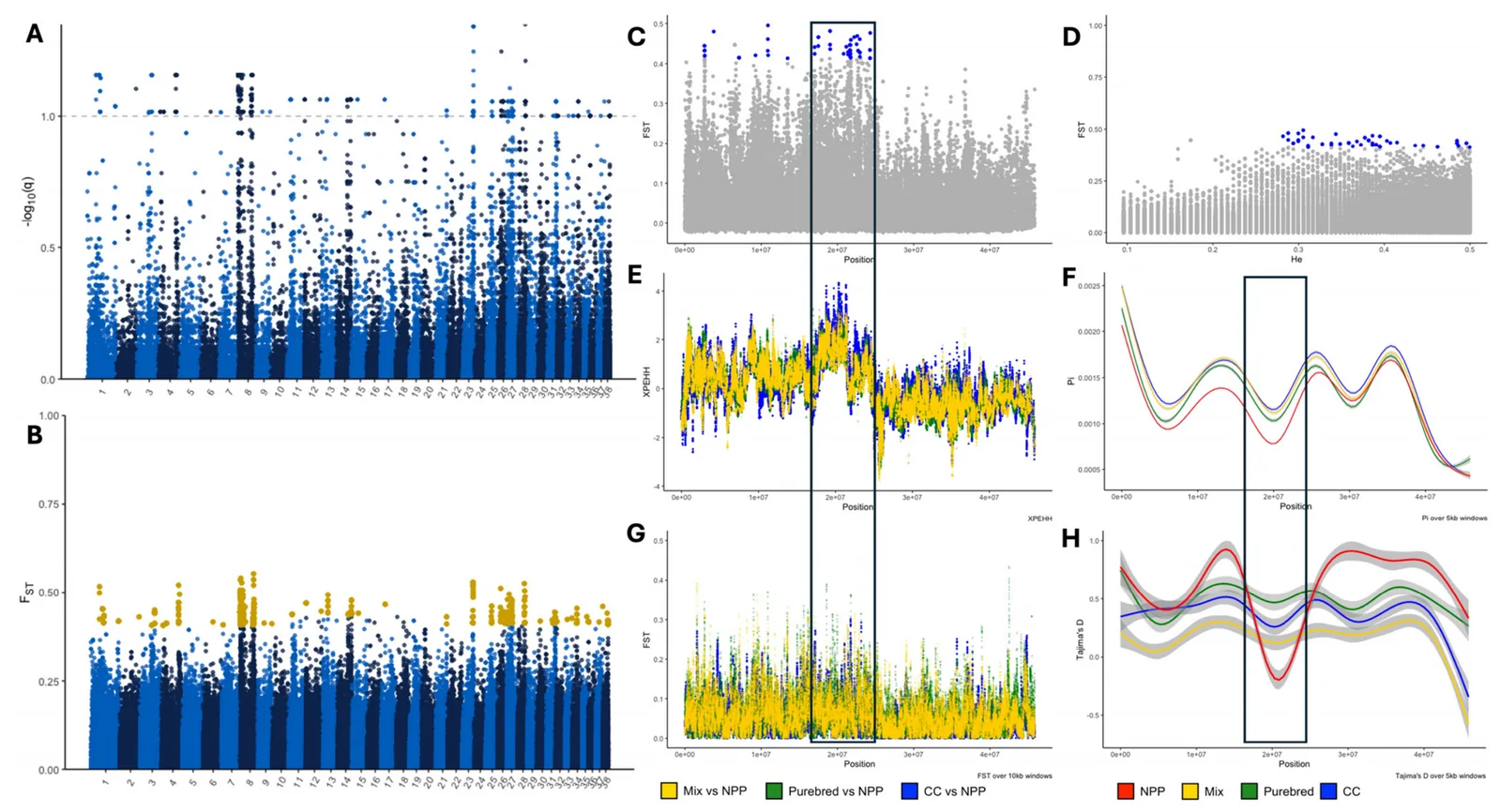
Results from the OutFLANK outlier analysis and plots of additional selection metrics for Chromosome 27. A Manhattan plot shows −log q-values for all analyzed loci across all autosomes, and the dashed horizontal line indicates the q-value threshold of 0.1 (**A**). A second Manhattan plot indicates the *F_ST_* estimates calculated by OutFLANK for each analyzed locus, with gold points highlighting the identified outlier loci (**B**). The right panels highlight specific metrics for a single chromosome, chromosome 27, as an example. Additional plots for all other autosomes that had outlier loci are included in Figure S3. Plots show FST estimates across positions (**C**) and expected heterozygosity estimates (**D**) for all considered loci in grey, with outlier loci highlighted in blue. Plots in (**E**–**H**) visualize the results of different calculations along the length of the chromosome, including XP-EHH (**E**), Pi along 5 kb sliding windows (**F**), FST along 10 kb sliding windows (**G**), and Tajima’s D along 5 kb windows (**H**). The position of one region of interest, including 61 outlier loci, is boxed throughout these plots. NPP is Nuclear Power Plant and CC is Chornobyl City.

**Table 1:** Effective population size (Ne) and Effective Number of Breeders (Neb) estimates from NeEstimator analysis.

				Molecular Coancestry				Linkage Disequilibrium							Heterozygote Excess				
Trial/ Population		Sample Size	Harmonic Mean	f1^	Neb^	CIs (Jack-Knife)		No. Indep. Allele Comparisons	r^2^	Expected (r^2^) Sample	Ne^	CIs (Jack-Knife)		Eff.df	No. Indep. Alelles	Weighted Mean D	Estimated Neb^	CIs (Parametric)	
Trial 1 (5000 loci)	CC	30	30.8	0.0874	5.7	5.3	6.2	11,165,175	0.0433	0.0369	46.1	26.7	114.3	87	4726	−0.02013	∞	∞	∞
	NPP	33	36.6	0.1062	4.7	4.5	5.0	9,669,003	0.0457	0.0332	24.5	16.1	40.8	612	4398	0.00243	206.6	62.8	∞
Trial 2 (5000 loci)	CC	30	30.8	0.1567	3.2	2.9	3.4	11,094,405	0.0437	0.0369	43.5	25	109.3	780	4690	−0.02463	∞	∞	∞
	NPP	33	36.5	0.1109	4.5	4.2	4.8	9,515,703	0.0451	0.0332	25.8	17	43.2	650	4413	0.00468	107.4	49.9	∞
Trial 3 (5000 loci)	CC	30	30.8	0.1138	4.4	4.1	4.7	11,132,121	0.0437	0.0369	43.5	24.4	117.8	705	4719	−0.02443	∞	∞	∞
	NPP	33	36.7	0.1010	5	4.7	5.2	9,832,395	0.0459	0.0332	24.1	15.7	40.7	58	4435	0.00224	223.4	65.1	∞
Trial 4 (Sep. by 100 kb)	CC	30	29.8	0.3405	1.5	1.4	1.5	151,164,552	0.0446	0.0372	39.9	22.3	105.8	633	17,410	−0.03923	∞	∞	∞
	NPP	33	32.4	0.1410	3.5	3.4	3.7	105,943,822	0.0465	0.0337	23.9	15.8	39.3	632	14,577	−0.00827	∞	∞	∞

Genome-wide results are shown across four trials and three methodologies, including Molecular Coancestry, Linkage Disequilibrium, and Heterozygosity Excess for each population. CC is Chornobyl City and NPP is Nuclear Power Plant. Parent-based coancestry (f1^), Effective population size (Ne^), Effective number of breeders (Neb^), Confidence Interval (CI), Effective degrees of freedom (Eff.df), Infinite (∞).

**Table 2:** Pairwise *F_ST_* results for the reduced density SNP dataset, including the comparison of free-breeding dog populations.

	CC	ERUS	GAHD	IS	PL	NPP	RUS	SC
**CC**		0.018	0.037	0.039	0.019	0.039	0.021	0.034
**ERUS**	0.018		0.025	0.027	0.006	0.051	0.006	0.022
**GAHD**	0.037	0.025		0.016	0.024	0.073	0.028	0.004
**IS**	0.039	0.027	0.016		0.026	0.077	0.028	0.014
**PL**	0.019	0.006	0.024	0.026		0.051	0.009	0.02
**NPP**	0.039	0.051	0.073	0.077	0.051		0.055	0.071
**RUS**	0.021	0.006	0.028	0.028	0.009	0.055		0.024
**SC**	0.034	0.022	0.004	0.014	0.02	0.071	0.024	

Estimates were generated for all pairs in the Nuclear Power Plant population, the Chornobyl City population, the Eastern European populations, and the Galápagos Archipelago populations. Shading scales from white (lowest *F_ST_*) to green (highest *F_ST_*). CC is Chornobyl City, ERUS is Eastern Russia, GAHD is Galápagos Hunting Dogs, IS is Isabela Island, PL is Poland, NPP is Nuclear Power Plant, RUS is Russia, and SC is Santa Cruz Island.

**Table 3:** Pairwise *F_ST_* results for the whole genome dataset.

	Purebred	Chornobyl City	Mix	Nuclear Power Plant
**Purebred**		0.029	0.014	0.068
**Chornobyl City**	0.029		0.022	0.052
**Mix**	0.014	0.022		0.063
**Nuclear Power Plant**	0.068	0.052	0.063	

Estimates were generated across all autosomes for all pairings amongst the Nuclear Power Plant population, the Chornobyl City population, the mixed-breed group, and the purebred group. Shading scales from white (lowest *F_ST_*) to green (highest *F_ST_*).

**Table 4:** Details for the 17 networks identified through the Ingenuity Pathway Analysis.

© 2000–2025 QIAGEN. All Rights Reserved.				
ID	Molecules in Network	Score	Focus Molecules	Top Diseases and Functions
1	26S proteasome, Ag-substrate:E3:E2:Ub, **BARD1**, **BAZ1A**, **CAPRIN2**, CG, **CPSF2**, E3 ligases in proteasomal degradation, E3:K48-iquitinated substrate, E3:Ub:substrate, EMC9, **FBLN5**, **FBXO21**, **FBXW8**, H3-3A/3B, **HECTD1**, histone, histone H2A, **MYEF2**, **NFKBIA**, **NGDN**, **NOP9**, **NOVA1**, p38 MAPK, RNA polymerase II, **RNMT**, **RPAP3**, **SELENOM**, **SRRM4**, **SUMO3**, **TIAL1**, **TRIP11**, **UBE2G2**, ubiquitin, **ZNF496**	42	22	DNA Replication, Recombination, and Repair, Embryonic Development, RNA Post-Transcriptional Modification
2	**ACIN1**, **ADARB1**, **AIRE**, alpha catenin, Ap1, **ARHGAP5**, **BBS9**, **CDH24**, CHMP4A, collagen type i (family), **DNMT3L**, **FABP4**, **FAM187B**, Ficolin-rich granule lumen proteins, **GCSAML**, **GPI**, **HEATR5A**, histone H3, histone H4, **HSPA8**, IKB, IKK (complex), IPO4, **LRRK2**, MAP2K1/2, **NEK6**, **NTAQ1**, **NYNRIN**, **PCK2**, **PFKL**, PI3K (family), PKA catalytic subunit, **PRMT5**, **RBM23**, Secretory granule lumen proteins	39	21	Cancer, Cell Morphology, Cellular Function and Maintenance
3	**AKAP6**, AKT, **AMIGO1**, **BDH1**, Calcineurin (complex), calmodulin, calpain, CaMKII, **CHGA**, Collagen type IV, CREB, **EIF4EBP2**, **FXYD1**, G protein beta gamma core, **GPSM3**, GSK3, ITPR, **ITPR2**, **JPH4**, **KCND2**, L-type calcium channel, MEF2, **mir-208** (includes others), **MYH6**, Myosin, **NEFH**, **NFATC4**, NMDA receptor, **NRL**, PKG, PP2A, PRKD, **PRKG2**, **PTTG1IP**, **SLC24A4**	32	18	Cardiovascular System Development and Function, Cellular Assembly and Organization, Molecular Transport
4	**ABHD4**, actin, **APCS**, **ATP5PD**, ATPase, C1Q, caspase, CD3 (complex), **CMTM5**, Collagen type I (complex), cytokine, **DHRS4**, IFN gamma, IgG2b, Laminin (complex), LDH (complex), **MEGF10**, MEK, MHC II, **MYH7**, Nalp3 inflammasome, NFAT (family), **NLRP3**, **PPP2R3C**, **RABGGTA**, **RIPK3**, **RNF185**, **RNF5**, **ST6GAL1**, STAT5A/B, TCR (complex), **TFCP2**, **TM9SF1**, TNF (family), **TRPM2**	29	17	Cellular Function and Maintenance, Infectious Diseases, Organismal Injury and Abnormalities
5	Cornified envelope, Cornified envelope:CDSN, cytokeratin, **DLGAP2**, Early cornified envelope, FLG fragment:Keratin tonofilament:Desmosome, Immunoglobulin, **INPP5J**, keratin, Keratin associated proteins, Keratin filament units, Keratin heterodimer, keratin II, 6, Keratin tonofilament:Desmosome, Keratin tonofilament:Keratin associated proteins polymer, Keratin type II, KRT5, KRT5/6, **KRT6A**, **KRT6B**, **KRT71**, **KRT74**, **KRT75**, **KRTAP10-1**, **KRTAP10-5**, **KRTAP10-7**, **KRTAP10-8**, **KRTAP12-3**, Late cornified envelope, Mature cornified envelope, **NODAL**, NOTCH, **NOTCH4**, TEAD, **TGM1**	27	16	Cellular Compromise, Cellular Development, Dermatological Diseases and Conditions
6	Adaptor, Adaptor protein 1, **ANO3**, **ANXA13**, **AP1B1**, **AP1G2**, **ARF1**, **CA10**, chemokine, **CIDEB**, **DAD1**, FcER1, **FCER1A**, **HIGD1C**, **ICOSLG**, IFN beta, IFN type 1, IFNAR, IgA, IgE, IgG, IgM, IL12 (complex), IL12 (family), **IL25**, interferon alpha, IRF9, NEDD8-MDP1, NFkB (complex), **PDE10A**, RNF31, **SINHCAF**, **SOX5**, TLR, **TTC28**	25	15	Cell Morphology, Cellular Assembly and Organization, Inflammatory Response
7	20S proteasome, 26S proteasome Reactome, 26S proteasome Reactome b, ADRM1:26S proteasome, ADRM1:26S proteasome:UCHL5, ADRM1:26S proteasome:USP14, **ATXN3**, **DPPA4**, **DUSP23**, **ITPK1**, JNK, KHNYN, **LETMD1**, **LHX6**, **MBIP**, MDP1, NEDD8, NEDD8, UBD:NUB1:26S proteasome, NEDD8-AcM-UBE2M:CRL1 E3 ubiquitin ligase:COMMDs:CCDC22:DCUN1Ds, PA28/20S immunoproteasome, **PALD1**, **PCBP3**, phosphatase, **PPP4C**, **PRORP**, proteasome, **PSMA6**, **PSMB5**, PSME1, PSME2, REC8, SOD, TBX6:RIPPLY2:26S proteasome, **THTPA**, YAP/TAZ	23	14	Cell Morphology, Cellular Function and Maintenance, Nucleic Acid Metabolism
8	**AGER**, ALP, **BMP3**, **CEBPE**, collagen alpha1, cytochrome-c oxidase, **DCAF11**, ERK, ETFBKMT, Fc gamma receptor, HDAC, HSP27, HSP70, HSP90 (family), IL1, LDL, Mitochondrial ETC 1, NADPH oxidase, **NDUFA8**, **NDUFV3**, NGF, NOS, **NOS1**, **NOS1AP**, **NPAS3**, **NUBPL**, **PBX1**, **PBX2**, PDGF-BB, **PKNOX1**, proinsulin, RB, **SMTN**, TGF beta, TSSK4	23	14	Cardiovascular Disease, Congenital Heart Anomaly, Developmental Disorder
9	**ABCG1**, **ACKR1**, activin (family), **AGPAT1**, CMA1, CPLA2, creatine kinase, **CRP**, CTSG, ECM, ENaC, ERK1/2, estrogen receptor, **FXYD5**, growth hormone, GTPase, HDL, histone deacetylase, **HTRA3**, IgG2c, IL23, lysosomal protease, N-CoR, Na, K-ATPase, **NELL2**, NR1H, **PRLR**, pro-inflammatory cytokine, **RGS10**, SAA, serine protease, **SIK1**, **SLC7A8**, **SSPN**, **TESC**	21	13	Cancer, Cardiovascular Disease, Cellular Development
10	14-3-3, ADCY, **ADCY4**, **AJUBA**, AMPK, BCR (complex), EGLN, Fibrinogen, FSH, G protein alpha i, **GNAI3**, GPCR, **GPR61**, **HAUS4**, IKKA/B, insulin, **KCNJ3**, **KSR2**, LH, **LTB4R2**, MAPK, NFkB (family), p70 S6k, **PITPNB**, PKA, PKC, PLC, PLC gamma, **PRKD1**, RAC, **REM2**, SRC (family), **SUCNR1**, TINF2, VEGF	19	12	Dermatological Diseases and Conditions, Developmental Disorder, DNA Replication, Recombination, and Repair
11	acetate, **ANKRD7**, asialo GM1 ganglioside, ATF6, C1QTNF4, **CBLN3**, CEBPD, **CFAP410**, cryptochrome, **CSRNP2**, ER/estrogen, ESR1, ESR1/FOS/JUN/estrogen, ESTG:ESR1 dimer:ATF2:JUN, ESTG:ESR1 dimer:JUN:FOS, **FAM177A1**, **FHAD1**, GCR/Pkac/RELA/glucocorticoid, IgG1, JUN, miR-519d-5p (miRNAs w/seed CUCCAAA), MITA1, N-docosahexaenoylethanolamine, NEDD9, NEK5, NR3C1, PRKAA, RELA, **SH3TC1**, **SLC5A12**, **TC2N**, **UMODL1**, YWHAB, **ZBTB21**, ZNF665	17	11	Cell Cycle, Cell Death and Survival, Gene Expression
12	ADAM20, Adam21, Adam24, Adam25 (includes others), Adam26a/Adam26b, **ADAM29**, Adam32, Adam4/Gm4787, Adam5, Adam6, Adam6a/Adam6b, ADAMTS19, APP, ATP1A1, Axonal Guidance Signaling, Brcc3, Brcc3dc, CMTM1, **CPNE6**, **DBX2**, **DHRS1**, **FITM1**, **FLRT2**, **FXYD3**, HRAS, IL1B, monosialoganglioside, MRPL12, **PLA2G3**, SDR39U1, **SFTA3**, **SLC11A2**, SMPD1, SOS, **TMEM51**	17	11	Cell Cycle, Lipid Metabolism, Small Molecule Biochemistry
13	ACCS, ACTB, ANLN, ARHGAP35, **ATXN7L2**, **CARMIL3**, **CATSPERB**, CBS, CCDC110, CFAP47, CFTR, **CYB561D1**, DENND5B, EFCAB11, FAM186A, H4C1, HSPA8 bound autophagic substrates, HSPA8:Substrate:LAMP2a, **LGI4**, **LSM8**, MSH4, Nlrp1b, Poly-vimentin:Misfolded Proteins:HDAC6, **RASSF8**, **RBM18**, **RNF212B**, SHOC1, SNRPD2, **STON2**, Substrate:LAMP2a, Substrates for chaperone mediated autophagy, UBR3, VIM, XKR6, **ZNF112**	17	11	Endocrine System Disorders, Organismal Injury and Abnormalities, Reproductive System Disease
14	**AGXT2**, **AK3**, **BCAT1**, beta-estradiol, CCDC17, CPA2, CPA5, **CPO**, **DACT2**, Gzmc, GZMH, HSPA5, ISM1, Klre1, L-glutamic acid, LINC00662, **LRRC20**, MDFIC, MFRP, miR-193a-3p (and other miRNAs w/seed ACUGGCC), MNX1-AS1, **MORN5**, mt-Atp8, Myh8, PAQR7, SHOC1, SLC17A1, SLC17A8, **SLC22A3**, SLC25A38, STAT3, TRIM54, **TSPEAR**, WNT3A, **ZFHX2**	15	10	Cancer, Cellular Development, Cellular Growth and Proliferation
15	EBF1:LDB1:LHX2:Intergenic olfactory enhancer:Olfactory Receptor gene, odorant:Olfactory receptor, odorant:Olfactory Receptor:GNAL:GDP:GNB1:GNG13, odorant:Olfactory Receptor:GNAL:GTP:GNB1:GNG13, Olfactory Receptor (characterized), Olfactory Receptor Gene, Olfactory Receptor Reactome, OR10J4, **OR10J5**, OR1E3, OR1F12P, OR2A1, OR2AP1, **OR2B11**, **OR2C3**, OR2D2, OR2D3, **OR2G3**, **OR2H1**, OR2I1P, OR4C46, **OR4L1**, OR51A7, OR51H1, OR51J1, OR52E2, OR52J3, OR52M1, OR52Z1P, OR5D16, **OR6B1**, **OR6C4**, **OR6F1**, OR8G2P, OR8I2	15	10	Cancer, Cell-To-Cell Signaling and Interaction, Nervous System Development and Function
16	43S translation preinitiation, 48S ribosome, AKT1, **AMN1**, ARHGAP24, **ARMC2**, BEST4, BGLT3, **CADM3**, DUXAP8, EIF1A (family), EIF4A/EIF4E/EIF4Γ, ESR2, Gm15483, Gm19378, GMPR2, **GOLGA5**, KCNK15-AS1, **KLHL38**, LINC01016, LOC100365839, miR-718 (miRNAs w/seed UUCCGCC), MITF, MYC, **NKX2-8**, **PAX9**, PTEN, Ribosome 40S, Ribosome 80S, Rn18s-rs2, Rn18s-rs6, RNA18SN5, Rps4y2, **SLC24A5**, UBE3A	11	8	Cancer, Cell Cycle, Cellular Development
17	18SE pre-40S particle, 40S ribosomal, 40S ribosome/eIF3/mRNA/eIF4A/eIF4B/eIF4E/eIF4G, 40S:eIF3:eIF1A, 43S, 43S: Ceruloplasmin mRNA:eIF4F:eIF4B:eIF4H:PABP, 43S:mRNA:eIF4F:eIF4B:eIF4H, 80S Ribosome:mRNA:peptidyl-tRNA with elongating peptide, 80S:aminoacyl tRNA:mRNA:eEF1A:GTP, 80S:Met-tRNAi:mRNA:eIF5B:GTP, 80S:Met-tRNAi:mRNA:SECISBP2:Sec, 80S:Met-tRNAi:mRNA:SECISBP2:Sec-tRNA(Sec):EEFSEC:GTP, GCN1:80S ribosome:mRNA, IMPACT:GCN1:80S ribosome:mRNA, L13a associated wth the 3 prime UTR GAIT element of ceruloplasmin mRNA within the translation initiation, Me-Q185-ETF1 dimer:eRF3:GDP:80S Ribosome:mRNA:peptidyl-tRNA, Me-Q185-ETF1 dimer:eRF3:GTP:80S Ribosome:mRNA:peptidyl-tRNA, **MTHFD2**, Phosphorylated UPF1:SMG5:SMG7:SMG6:PP2A:Translated mRNP, PI3K (complex), polypeptide signal:Translocon, Ribosome/HBS1L/PELO, **RP1L1**, **RPL36A**, **RPS4X**, **RPS4Y1**, **RPS4Y2**, SMG1:UPF1:EJC:Translated mRNP, SRP receptor:SRP:ribosome:polypeptide signal, **SRP54**, SRP:polypeptide signal:ribosome, Translated mRNA Complex with Premature Termination Codon Not Preceding Exon Junction, Translated ROBO3.2 mRNA in complex with NMD-initiating UPF2, tRNA:EIF2AK4:GCN1:80S Ribosome:mRNA, UPF1:eRF3 Complex on Translated mRNA	9	7	Connective Tissue Disorders, Developmental Disorder, Hereditary Disorder

The identified gene dataset that served as the input for IPA includes genes located within 50 kb of the genomic regions identified through the OutFLANK outlier analysis. Genes highlighted as part of our dataset are bolded.

## Data Availability

All data used and analyzed during this study are available through their respective referenced publications. The main WGS dataset for the Chornobyl dogs was previously made available through NCBI BioProject PRJNA1087248.

## Supplementary Materials

The following supporting information can be downloaded at: https://www.mdpi.com/article/10.3390/genes17101195/s1, Figure S1: Principal Component Analysis (PCA) for groups within the whole genome sequence dataset; Figure S2: Deviation in per chromosome *F_ST_* compared to the pairs’ all-autosomal estimate; Figure S3: Detailed metrics across autosomes that contain outlier loci as identified through OutFLANK; Figure S4: Top associated diseases summary from the Ingenuity Pathway Analysis; Table S1: Summary of effective population size (Ne) estimates from NeEstimator analysis per chromosome; Table S2: Positional information and results from subsequent analysis for the identified OutFLANK outlier regions.

## Abbreviations

The following abbreviations are used in this manuscript:

CEZ	Chornobyl Exclusion Zone
NPP	Nuclear Power Plant
CC	Chornobyl City
WGS	Whole Genome Sequence
DAPC	Discriminant Analysis of Principal Components
PCA	Principal Components Analysis
