# Supplementary material for: Evidence of Selective Response in the Free-Breeding Dog Population in Chornobyl
Source: Genes (Basel). 2026 Sep 27;17(10):1195. doi: 10.3390/genes17101195 (PMC13704518; doi:10.3390/genes17101195)
Supplement: Supplementary file 1 [file genes-17-01195-s001.zip › genes-4559477-supplementary.pdf]

Table S1. Summary of effective population size (Ne) estimates from NeEstimator analysis per chromosome. Results are shown across three methodologies, including, Linkage Disequilibrium (LD), Heterozygosity Excess (Het), and Molecular Coancestry (CN) for all 38 autosomes and for each population. CC is Chornobyl City and NPP is Nuclear Power Plant.

Chromosome	Pop	LD	Het	CN
1	CC	19.4	infinite	1.8
1	PP	5.7	infinite	3.1
2	CC	21.9	infinite	2.4
2	PP	3.6	infinite	2.7
3	CC	17.3	infinite	1.4
3	PP	3.1	infinite	2.1
4	CC	16.1	infinite	4.8
4	PP	4.1	166.9	2.4
5	CC	21	infinite	2.5
5	PP	7.9	12.8	2.2
6	CC	10.6	infinite	4.3
6	PP	3.6	49.2	3.9
7	CC	16.8	infinite	3.1
7	PP	3.9	infinite	2.7
8	CC	15.7	infinite	5.2
8	PP	3.2	260.3	2.3
9	CC	18.4	infinite	2.5
9	PP	3.7	infinite	1.1
10	CC	21.7	infinite	1.6
10	PP	6.7	infinite	2.6
11	CC	22.2	infinite	3.4
11	PP	3.3	infinite	2.6
12	CC	13.7	infinite	3.1
12	PP	6.5	17.5	2.8
13	CC	16.1	infinite	3
13	PP	3.5	infinite	3.5
14	CC	12.2	infinite	2.8
14	PP	3.4	34.9	3
15	CC	14.6	infinite	2.7
15	PP	3.7	13	2.2
16	CC	12.3	infinite	3.7
16	PP	5.9	25	3.6
17	CC	15.6	infinite	3.3
17	PP	6.3	infinite	3.2
18	CC	14.8	infinite	2.5
18	PP	3.9	infinite	3.2
19	CC	11.1	infinite	2.1
19	PP	3.3	infinite	1.7
20	CC	16.4	infinite	3.1
20	PP	3.4	2346.1	2.7
21	CC	15.6	infinite	2.3
21	PP	3.2	infinite	2.8
22	CC	15.6	infinite	1.9
22	PP	2.7	infinite	2.1
23	CC	15	infinite	4.5
23	PP	3.3	21.3	2.9
24	CC	17.5	infinite	3.7
24	PP	3.9	13.3	3.9
25	CC	16.4	127.6	3.7
25	PP	3.7	infinite	1.8
26	CC	15.5	infinite	3.5
26	PP	2.9	50.4	2.7
27	CC	19.2	infinite	4.8
27	PP	6.2	infinite	1.8
28	CC	18.8	infinite	2.3
28	PP	3.1	infinite	1.8
29	CC	17.9	infinite	5.3
29	PP	3	infinite	2.8
30	CC	15.5	infinite	2.4
30	PP	3	infinite	2
31	CC	19.1	infinite	1.4
31	PP	3.7	11	3.3
32	CC	17.5	80.9	1.4
32	PP	5.2	168.8	2.7
33	CC	11.2	infinite	3.1
33	PP	3.3	86.1	3.3
34	CC	18.3	infinite	3.9
34	PP	3.6	infinite	1.8
35	CC	17.5	1468	6.5
35	PP	3.8	infinite	3
36	CC	12	infinite	3.9
36	PP	4	11.8	2.4
37	CC	15.1	infinite	3
37	PP	3.1	infinite	1.4
38	CC	20	infinite	2.4
38	PP	3.7	infinite	2.6

Table S2. Positional information and results from subsequent analysis for the identified OutFLANK outlier regions. The chromosome, positions, number of loci in each region, distance covered by the region, identified genes, and overlap across additional analyses is included.

OutFLANK outlier info						XP-EHH Ratio (average per local peak)				Overlap for additional metrics?		
Chrom	Start	End	No.loci	Distance	Genes within 50 kb	Summary	CC vs NPP	Purebred vs NPP	Mix vs NPP	Sliding Window FST	Nucleotide Diversity	Tajima's D
1	36596866	36601409	3	4543		+ ratio	1.47	1.70	2.59	local peak in FST	local minima	high, negative value
1	49427295	49429007	8	1712	SLC22A3	+ ratio	0.53	0.24	0.19	local peak in FST	local minima	high, negative value
1	53714200	53740784	18	26584	PDE10A	+ ratio	0.79	0.53	1.36	local peak in FST	local minima	high, negative value
1	55772884	55774971	2	2087	DACT2	+ ratio	0.97	0.30	1.17	local peak in FST		
1	117413444	117422571	6	9127	FXYD1, LGI4, FXYD3, FXYD5, FAM187B	+ ratio	2.73	0.68	1.34	local peak in FST	local minima	high, negative value
2	82202636		1		FHAD1, TMEM51	+ ratio	0.21	0.74	0.74	local peak in FST	local minima	
3	50591314		1			+ ratio	2.41	1.2	1.02	local peak in FST	local minima	high, negative value
3	51021203		1			+ ratio	2.02	1.39	0.93	local peak in FST	local minima	high, negative value
3	60032011		1		SH3TC1, HTRA3	+ ratio	0.38	0.39	0.34	local peak in FST		
3	63260014	63273405	11	13391		+ ratio	0.47	0.3	0.35	local peak in FST	local minima	high, negative value
4	7369797	7380600	3	10803		+ ratio	1.10	1.33	1.13	local peak in FST		
4	21349488	21354421	4	4933	LRRC20, NODAL, PALD1, EIF4EBP2	+ ratio	1.74	1.71	1.71	local peak in FST	local minima	high, negative value
4	73226483	73261445	21	34962	PRLR, AGXT2	+ ratio	0.55	1.22	1.68	local peak in FST	local minima	high, negative value
4	77599046		1			+ ratio	3.49	2.00	1.69	local peak in FST	local minima	high, negative value
6	42327470		1		GNAI3, GPR61, AMIGO1, CYB561D1, ATXN7L2	+ ratio	0.39	0.30	0.30	local peak in FST	local minima	
7	7771600	7785168	2	13568		+ ratio	2.68	2.70	2.36	local peak in FST	local minima	high, negative value
8	38583	73696	6	35113						local peak in FST		
8	97783	200810	42	103027	ZNF496	+ ratio	1.15	0.41	0.65	local peak in FST	local minima	high, negative value
8	230534	1028596	359	798062	ZNF496, NLRP3, GCSAML, CATSPERB, OR6C4, OR6A6, OR2H1, OR2B11, OR2W6P, OR2C3, OR2G3, OR6F1, OR6B1, OR6C4, OR4L1	+ ratio	0.96	0.96	1.11	local peak in FST	local minima	high, negative value
8	1079246	1101857	3	22611	CATSPERB, TC2N	+ ratio	0.41	0.32	0.79	local peak in FST	local minima	
8	1145799	1190091	8	44292	TC2N, FBLN5	+ ratio	0.76	0.46	0.66	local peak in FST	local minima	
8	1224578	1585701	73	361123	FBLN5, TRIP11, ATXN3, CPSF2, SLC24A4	+ ratio	1.08	1.14	1.19	local peak in FST	local minima	
8	1907085	2125092	5	218007	GOLGA5, CHGA, ITPK1	+ ratio	1.02	0.36	0.65	local peak in FST	local minima	
8	2412300	2458075	2	45775	RNMT, BARD1					local peak in FST	local minima	
8	2776693	2979884	4	203191	DAD1, ABHD4, TCR-alpha chain C region	+ ratio	0.92	0.87	0.54	local peak in FST	local minima	high, negative value
8	3268285	3480653	17	212368	SLC7A8, REM2, PRMT5, AJUBA, RBM23, CDH24, PSMB5, ACIN1, CEBPE, RNF212B, HAUS4	+ ratio	1.19	0.92	0.91	local peak in FST	local minima	high, negative value
8	3664275	3746204	2	81929	IL25, NGDN, ZFHX2, THTPA, MIR208A, MYH6, MYH7, CMTM5, AP1G2, JPH4, MHRT	+ ratio	1.13	1.20	0.85	local peak in FST	local minima	high, negative value
8	3929479		1			+ ratio	1.29	0.88	0.71	local peak in FST	local minima	high, negative value
8	4079147		1		DHRS4, CARMIL3, CPNE6, NRL, PCK2, DCAF11, FITM1	+ ratio	1.22	1.32	1.04	local peak in FST	local minima	high, negative value
8	4277146	4350762	3	73616	TGM1, RABGGTA, DHRS1, NOP9, CIDEB, LTB4R2, RIPK3, ADCY4, NFCATC4, NYNRIN, CBLN3	+ ratio	1.59	1.59	1.16	local peak in FST	local minima	high, negative value
8	4572738		1			+ ratio	1.52	2.11	1.31	local peak in FST	local minima	high, negative value
8	5870338	6046820	24	176482		+ ratio	1.31	1.34	1.38	local peak in FST	local minima	high, negative value
8	6144661	6189402	2	44741		+ ratio	0.43	0.64	0.44	local peak in FST	local minima	
8	6312183	6387670	3	75487	NOVA1	+ ratio	0.98	1.34	1.18	local peak in FST	local minima	
8	6597901	7153036	27	555135	DPPA4, HSPA8, GPI, RPS4X, AK3, RPS4Y2, RPS4Y1	+ ratio	1.31	1.05	0.65	local peak in FST	local minima	high, negative value
8	8989224	9838166	16	848942	PRKD1	+ ratio	1.35	1.39	1.15	local peak in FST	local minima	high, negative value
8	10480338	10565017	5	84679	HECTD1, HEATR5A	+ ratio	0.86	1.13	0.84	local peak in FST	local minima	high, negative value
8	10966388	11380178	14	413790	NUBPL, ARHGAP5, AKAP6	+ ratio	2.03	2.68	2.80	local peak in FST	local minima	high, negative value
8	11859294	12172380	13	313086	AKAP6, NPAS3	+ ratio	1.98	2.06	1.90	local peak in FST	local minima	high, negative value
8	12404704	12569690	6	164986	NPAS3	+ ratio	2.01	1.68	1.77	local peak in FST	local minima	high, negative value
8	12987854	13153827	2	165973		+ ratio	1.85	2.23	1.95	local peak in FST	local minima	high, negative value
8	13700167	14057737	11	357570	SRP54, BAZ1A, FAM177A1, PSMA6, PPP2R3C, NFKBIA, PRORP	+ ratio	1.00	1.25	0.76	local peak in FST	local minima	
8	14873213	15176264	2	303051	MBIP, NKX2-8, PAX9, SFTA3	+ ratio	0.84	1.03	0.92	local peak in FST	local minima	high, negative value
8	20997681	21001055	6	3374		+ ratio	0.21	0.40	0.25	local peak in FST	local minima	
8	53681739	53683555	2	1816	STON2	+ ratio	0.15	0.75	0.90	local peak in FST		
8	54086144	54442625	17	356481	NEK6	+ ratio	1.14	1.17	1.28	local peak in FST	local minima	
8	55914655	55922454	13	7799		+ ratio	0.91	1.33	1.35	local peak in FST	local minima	
8	56205490		1			+ ratio	1.11	1.36	1.27			
8	56546001	56713761	5	167760		+ ratio	1.07	1.49	1.03	local peak in FST	local minima	high, negative value
8	56896105		1			+ ratio	1.49	1.86	1.45	local peak in FST	local minima	high, negative value
8	57011254	57027424	36	16170		+ ratio	1.01	1.70	1.00	local peak in FST	local minima	high, negative value
8	57172871	57276496	5	103625	FLRT2	+ ratio	1.74	2.05	2.05	local peak in FST	local minima	high, negative value
8	58302111	58550620	22	248509		+ ratio	1.44	1.33	1.28	local peak in FST	local minima	high, negative value
9	27525471		1		CA10	+ ratio	1.45	0.75	1.33	local peak in FST	local minima	high, negative value
9	60370421		1		RBM18, MORN5, NDUFA8, LHX6	+ ratio	0.92	0.73	0.73	local peak in FST	local minima	
11	16545801	16559743	10	13942	MEGF10	+ ratio	1.86	1.89	2.94	local peak in FST	local minima	high, negative value
12	1612815	1618175	4	5360	AGER, AGPAT1, RNF5, GPSM3, PBX2, NOTCH4, BTNL1	+ ratio	0.82	0.79	0.68	local peak in FST	local minima	high, negative value
12	65807555		1		ARMC2	+ ratio	1.55	2.11	1.14	local peak in FST	local minima	high, negative value
13	21955048	21968303	12	13255	ANXA13, KLHL38, WDYHV1	+ ratio	1.98	0.95	1.99	local peak in FST		
14	45609731	45651266	4	41535	BBS9					local peak in FST	local minima	high, negative value
14	56946307		1		LSM8, ANKRD7	+ ratio	1.89	1.64	2.17	local peak in FST	local minima	high, negative value
14	58732531	58780062	8	47531	KCND2	+ ratio	1.29	2.57	1.82	local peak in FST	local minima	high, negative value
14	59669310	59669529	3	219		+ ratio	0.24	1.71	0.81	local peak in FST		
15	27203499	27208658	2	5159		+ ratio	0.69	1.90	1.28	local peak in FST	local minima	high, negative value
17	17705144	17705255	2	111		+ ratio (Breed, Mix)		1.45	0.78	local peak in FST	local minima	high, negative value
21	47443240	47448203	3	4963	ANO3, SLC5A12	+ ratio	0.77	0.68	0.74	local peak in FST	local minima	
23	46442441	46449244	9	6803	SUCNR1	+ ratio	1.20	1.95	2.06	local peak in FST	local minima	
23	47681642		1			+ ratio	2.19	2.41	2.79	local peak in FST	local minima	high, negative value
23	47769337	47817960	16	48623		+ ratio	1.89	2.36	1.59	local peak in FST	local minima	
25	25547013		1		ADAM29	+ ratio	0.62	0.43	0.53	local peak in FST	local minima	
25	26866948	26978492	6	111544	XKR6					local peak in FST	local minima	high, negative value
25	27328558		1		RP1L1					local peak in FST	local minima	
26	12714951		1							local peak in FST	local minima	high, negative value
26	13597112	13960696	6	363584	NOS1, FBXO21, KSR2, FBXW8, TESC	+ ratio	1.54	1.27	1.47	local peak in FST	local minima	high, negative value
26	15178543		1		SRRM4	+ ratio	0.73	0.99	0.69	local peak in FST	local minima	
26	21386301	21399619	10	13318	PITPNB, TTC28					local peak in FST	local minima	high, negative value
26	22698340		1		NEFH, AP1B1, ATP5PD	+ ratio	1.17	1.78	1.01	local peak in FST	local minima	high, negative value
26	24110054		1		SELENOM, SMTN, RNF185, INPP5J, PLA2G3	+ ratio	1.71	1.30	1.39	local peak in FST	local minima	
26	31855197	31855337	2	140		+ ratio	0.79	0.73	0.83	local peak in FST		
27	2571625	2602726	11	31101	KRT5, KRT74, KRT71, KRT75, KRT124, KRT6A, KRT6B	+ ratio	0.87	1.32	0.72	local peak in FST	local minima	high, negative value
27	3788290		1		TFCP2, CSRNP2, LETMD1, SLC11A2, HIGD1C	+ ratio	0.96	1.06	1.31	local peak in FST	local minima	high, negative value
27	7105628	7106292	6	664	RPAP3, MTHFD2					local peak in FST		high, negative value
27	9263742		1		DBX2, NELL2	+ ratio	1.68	2.22	2.05	local peak in FST	local minima	high, negative value
27	10871534	10916978	4	45444	PPP4C	+ ratio	0.66	0.90	0.97	local peak in FST	local minima	
27	13468769		1		LRRK2	+ ratio (CC, Breed)	0.39	0.63		local peak in FST		
27	17015675	17016390	2	715	AMN1							
27	17489774	17503043	3	13269	CAPRIN2, SINHCAF	+ ratio	1.71	1.21	1.01	local peak in FST		high, negative value
27	19031206	19050311	5	19105		+ ratio	2.55	1.99	2.31	local peak in FST		high, negative value
27	20813654		1		ITPR2	+ ratio	2.44	2.60	1.90	local peak in FST	local minima	high, negative value
27	21167388		1		ITPR2	+ ratio	1.61	1.91	1.67	local peak in FST	local minima	high, negative value
27	21396304	21405083	3	8779	SSPN	+ ratio	3.15	2.16	2.55	local peak in FST	local minima	high, negative value
27	21636581	21699685	15	63104	RASSF8	+ ratio	0.88	0.87	1.08	local peak in FST	local minima	high, negative value
27	21720889	21764190	10	43301	RPL36A	+ ratio (except MIX)	0.13	0.87		local peak in FST	local minima	high, negative value

27	22201693		1							local peak in FST	local minima	high, negative value
27	22515524		1		FABP4	+ ratio	1.03	0.85	0.41	local peak in FST	local minima	high, negative value
27	22601159		1		BCAT1					local peak in FST	local minima	high, negative value
27	22707726	22717796	3	10070	RPL21P44	+ ratio	1.57	0.87	1.01	local peak in FST		high, negative value
27	22927450	22948870	4	21420	SOX5	+ ratio	1.28	1.02	0.61	local peak in FST		high, negative value
27	24264081	24296668	10	32587		+ ratio	1.56	0.98	0.42	local peak in FST	local minima	high, negative value
28	29706155	29706259	2	104	RGS10, TIAL1	+ ratio	1.83	2.55	2.57	local peak in FST		
28	30205121	30209534	11	4413	ARF1	+ ratio	0.41	0.75	0.61	local peak in FST	local minima	high, negative value
28	31059200	31062170	7	2970		+ ratio	1.26	1.46	0.72	local peak in FST	local minima	
28	32780642	32786607	2	5965		+ ratio (CC, Breed)	0.63	0.73		local peak in FST	local minima	high, negative value
30	14378412		1		MYEF2, SLC24A5	+ ratio	1.36	1.23	0.24	local peak in FST	local minima	high, negative value
31	24931327		1							local peak in FST	local minima	
31	27577667		1			+ ratio	0.18	1.06	0.91	local peak in FST	local minima	
31	36428526	36558583	8	130057	ZBTB21, ABCG1, UMODL1	+ ratio	2.06	1.74	1.68	local peak in FST	local minima	
31	37160231		1		NDUFV3, PKNOX1	+ ratio	1.61	1.42	1.18	local peak in FST	local minima	high, negative value
31	37436187	37460688	10	24501	SIK1	+ ratio	2.25	1.98	2.09	local peak in FST	local minima	
31	37491232	37491762	2	530	SIK1	+ ratio	1.67	1.63	2.31	local peak in FST	local minima	high, negative value
31	38121461	38183850	4	62389	DNMT3L, PFKL, TRPM2, CFAP410, ICOSLG, AIRE	+ ratio	1.61	0.95	1.24	local peak in FST	local minima	high, negative value
31	38363216		1		TSPEAR, KRTAP10-7	+ ratio	0.15	1.18		local peak in FST	local minima	high, negative value
31	38465455		1		SUMO3, UBE2G2, PTTG1IP, KRTAP10-5, KRTAP10-8, KRTAP12-3	+ ratio	0.88	1.97	0.63	local peak in FST	local minima	high, negative value
31	38747826		1		ADARB1, KRTAP10-1	+ ratio	0.89	1.07	1.36	local peak in FST	local minima	high, negative value
31	39059459		1		PCBP3	+ ratio	1.21	1.55	1.44	local peak in FST	local minima	
32	5225239	5226115	2	876	BMP3, PRKG2	+ ratio (CC)	1.35			local peak in FST		
33	10667996		1			+ ratio	0.49	0.37	0.37	local peak in FST	local minima	
33	30382055	30383633	2	1578	BDH1	+ ratio	2.02	1.73	1.08	local peak in FST	local minima	
34	19540301	19544087	3	3786	ST6GAL1	+ ratio	1.81	1.44	1.39	local peak in FST	local minima	high, negative value
34	20249399	20261702	5	12303		+ ratio	1.68	2.01	2.16	local peak in FST	local minima	high, negative value
35	20349121		1			+ ratio	0.94	1.18	0.57	local peak in FST	local minima	
36	1509771	1514312	11	4541	KCNJ3, MIR8797	+ ratio	0.20	0.60	0.75	local peak in FST	local minima	
37	15401218		1		CPO	+ ratio	0.22	0.36	0.33	local peak in FST	local minima	high, negative value
37	30802683		1		DLGAP2	+ ratio	0.40	0.88	0.67	local peak in FST	local minima	
38	18735547		1		PBX1	+ ratio	1.39	1.82	1.26	local peak in FST	local minima	high, negative value
38	20534743		1		NOS1AP	+ ratio	1.70	1.41	0.94	local peak in FST	local minima	
38	22392138	22422778	2	30640	CRP, DUSP23	+ ratio	2.77	3.04	2.78	local peak in FST	local minima	
38	22489042	22503665	4	14623	APCS, OR12E11	+ ratio	1.33	2.40	1.95	local peak in FST	local minima	
38	22552976	22627396	7	74420	OR10J5, OR10J4	+ ratio	1.94	2.74	2.63	local peak in FST	local minima	
38	22726256	22747000	6	20744	ACKR1, FCER1A, OR10J5, CADM3	+ ratio	1.71	2.30	2.77	local peak in FST	local minima	

Figure S1. Principal Component Analysis (PCA) for groups within the whole genome sequence dataset. PCA was conducted with 30 principal components. The x-axis is the first principal component (PC1), and the y-axis is PC2. Eigen values are included in the top left. NPP is Nuclear Power Plant, CC is Chornobyl City, Mix is Mixed Breed, and Breed is the Purebred group.

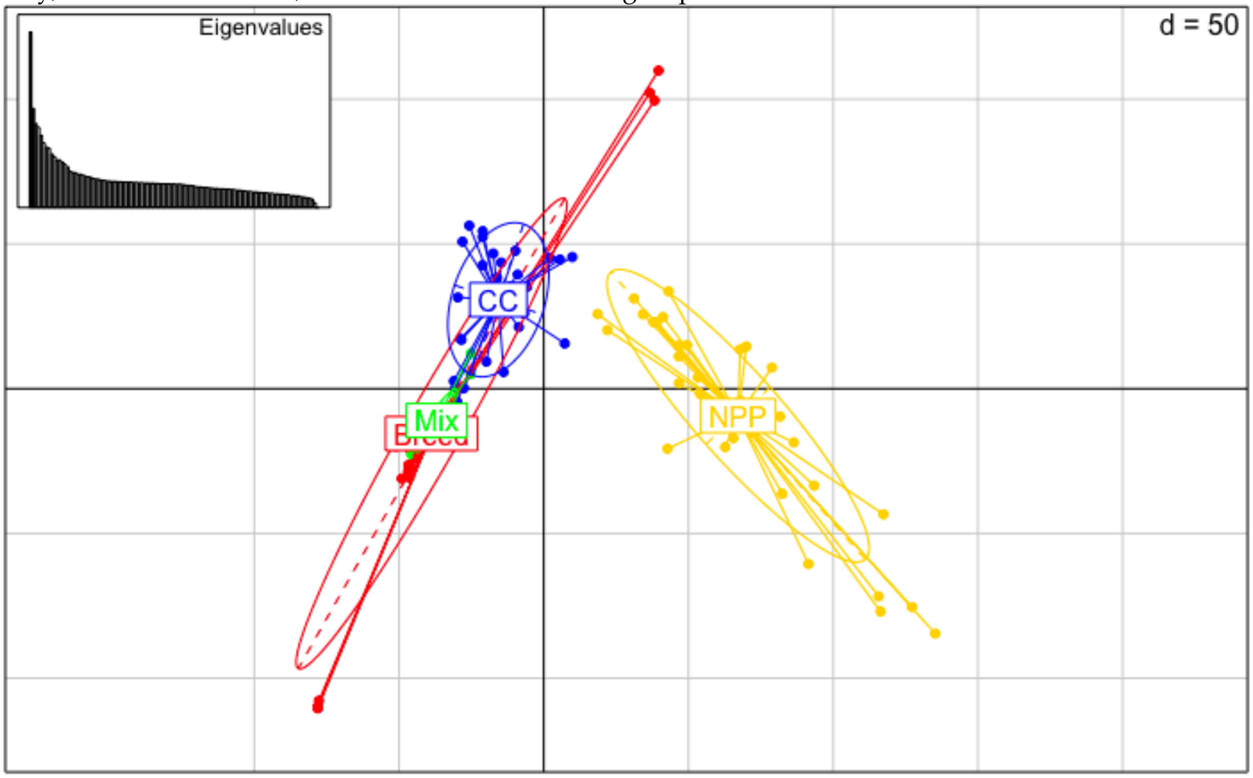

Figure S2. Deviation in per chromosome *F_{ST}* compared to the pairs' all-autosomal estimate. Each dot represents the deviation in FST for a single chromosome, and the box and whisker plot displays the standard Tukey-style data summary for each pair considered. Pairings that do not include the NPP are in silver, and those that include the NPP are in gold.

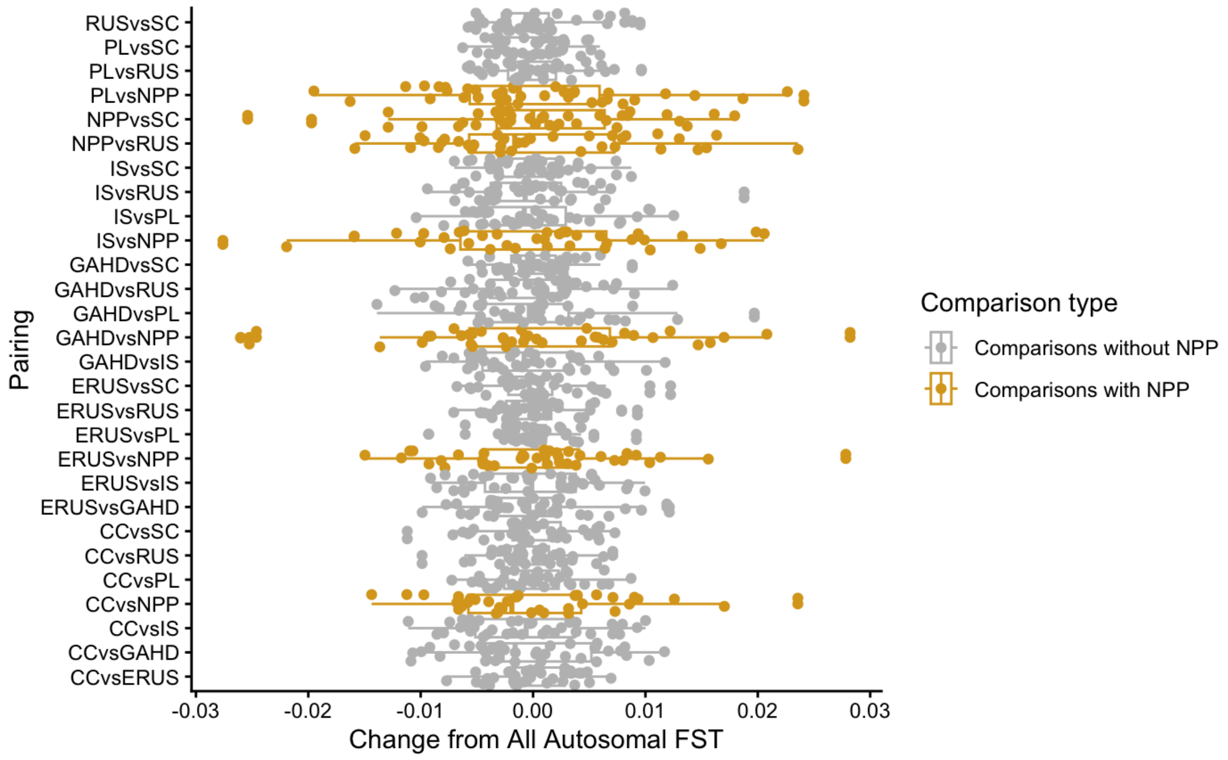

Figure S3. Detailed metrics across autosomes that contain outlier loci as identified through OutFLANK. The first panels include plots for *F_{ST}* estimates compared to expected heterozygosity for all considered loci in grey, with outlier loci indicated in blue (A). Each subsequent panel contains results for each chromosome along the length of the chromosome, including *F_{ST}* estimates from OutFLANK, XP-EHH, Pi along 5 kb sliding windows, *F_{ST}* along 10 kb sliding windows, and Tajima's D along 5 kb windows. The regions containing outlier loci are indicated with boxes throughout the plots.

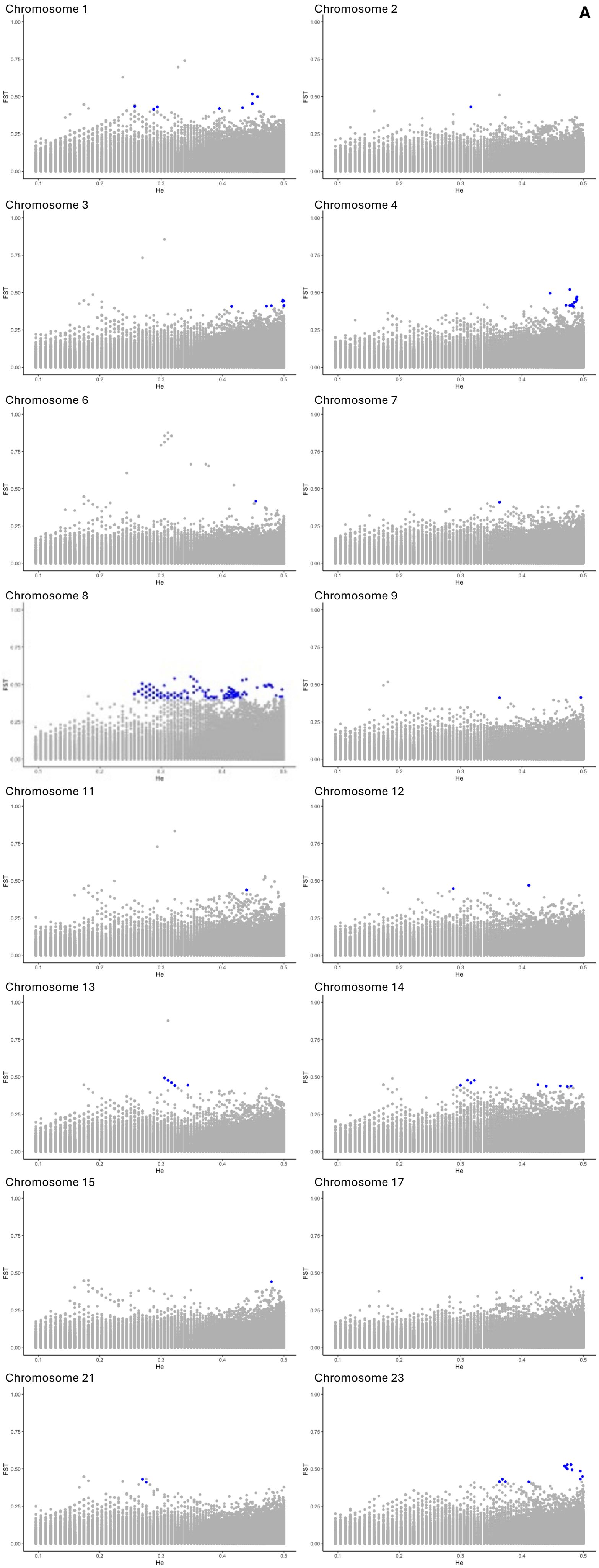
A
Chromosome 1
Chromosome 2
Chromosome 3
Chromosome 4
Chromosome 6
Chromosome 7
Chromosome 8
Chromosome 9
Chromosome 11
Chromosome 12
Chromosome 13
Chromosome 14
Chromosome 15
Chromosome 17
Chromosome 21
Chromosome 23
FST
He

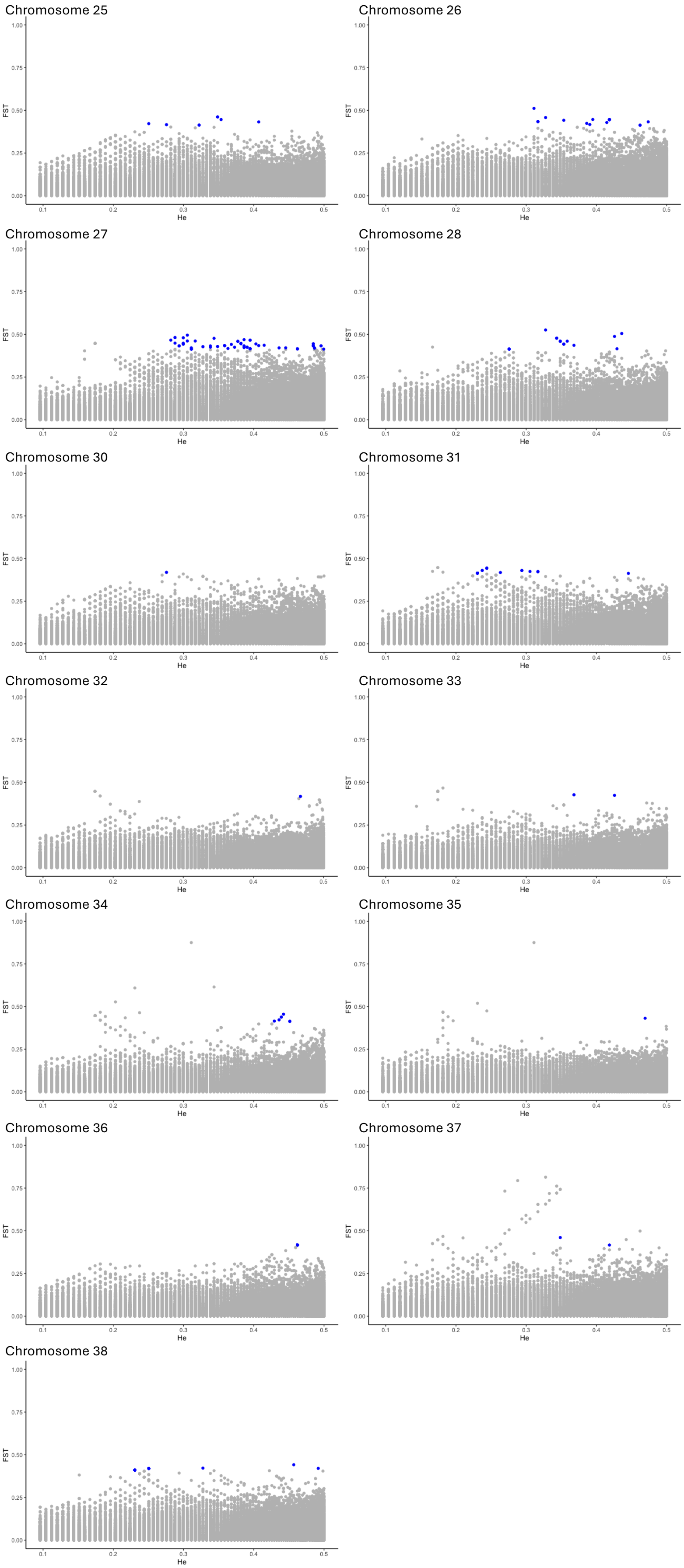

Chromosome 25
Chromosome 26
Chromosome 27
Chromosome 28
Chromosome 30
Chromosome 31
Chromosome 32
Chromosome 33
Chromosome 34
Chromosome 35
Chromosome 36
Chromosome 37
Chromosome 38
FST
He
0.00
0.25
0.50
0.75
1.00
0.1
0.2
0.3
0.4
0.5

Chromosome 1

B

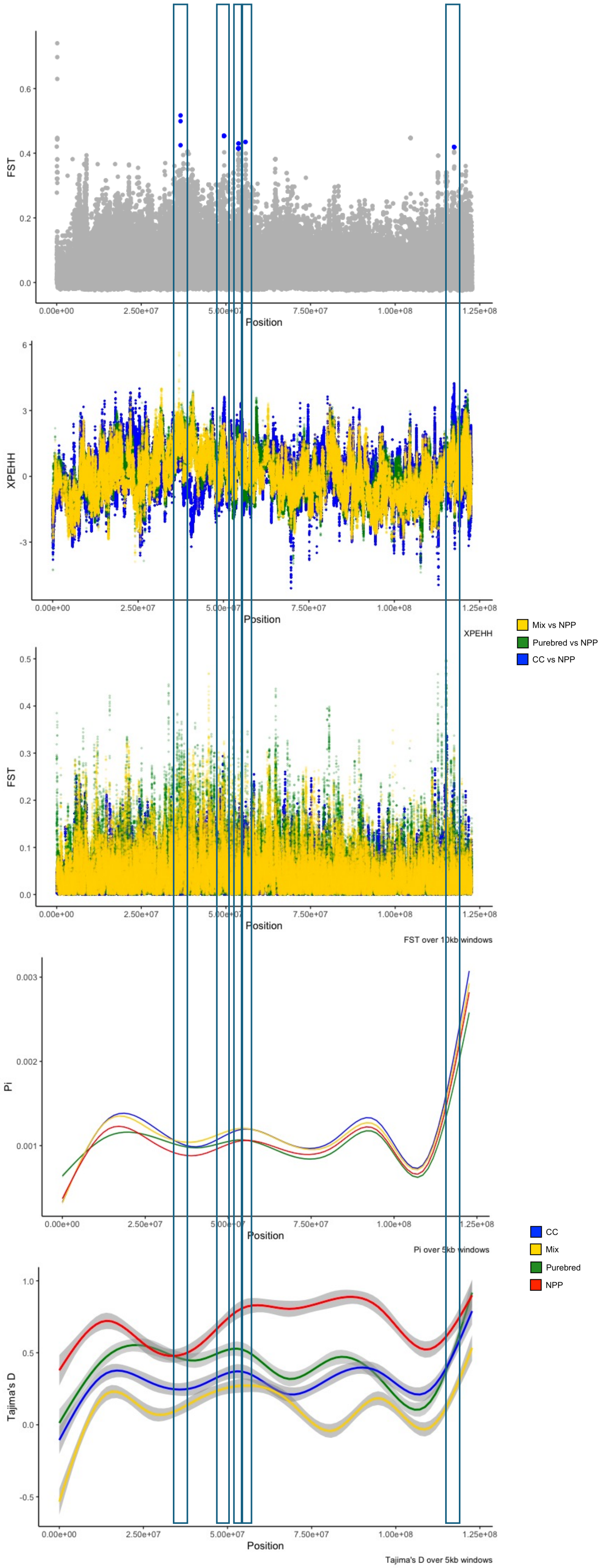

Chromosome 2

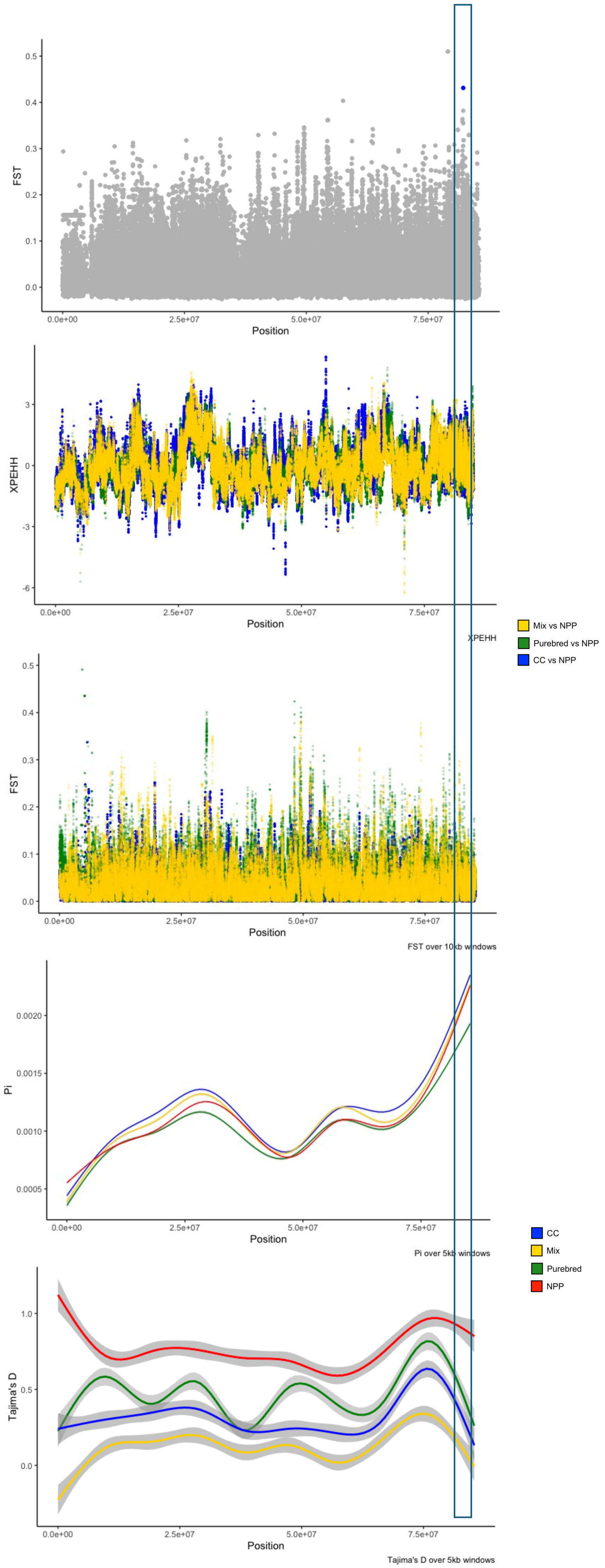

Chromosome 3

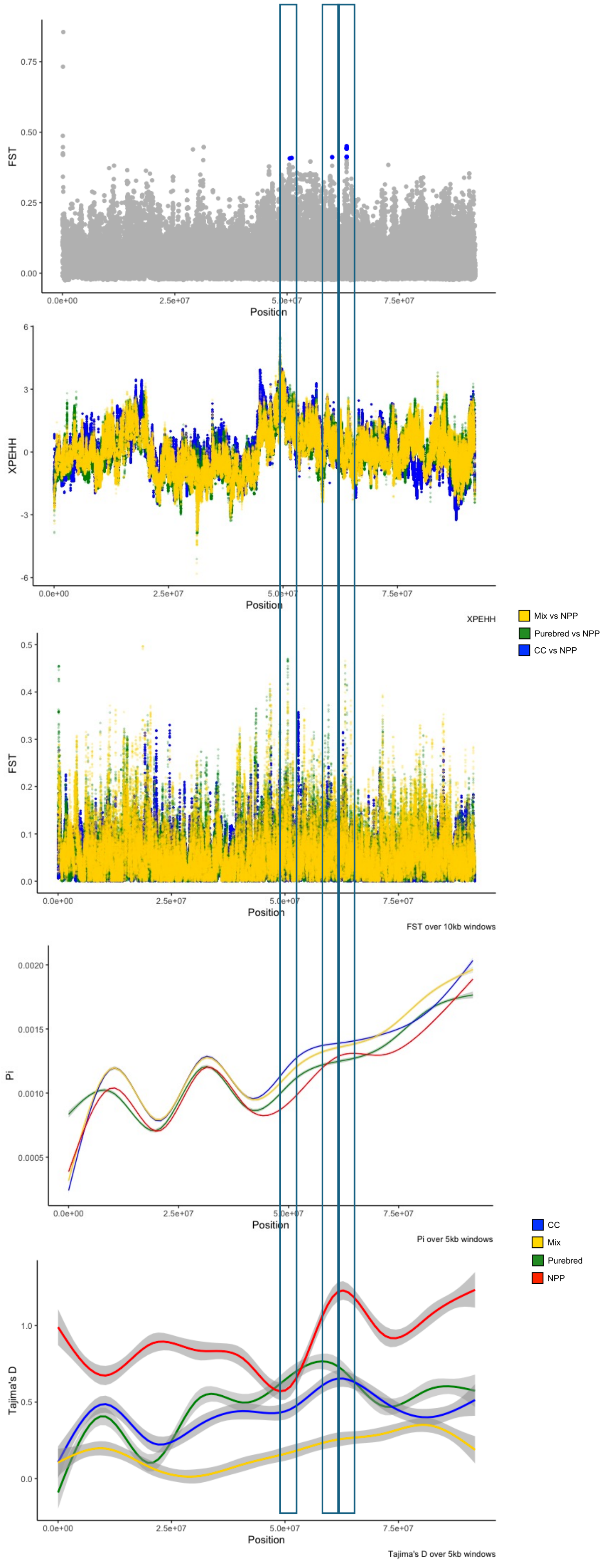

Chromosome 4

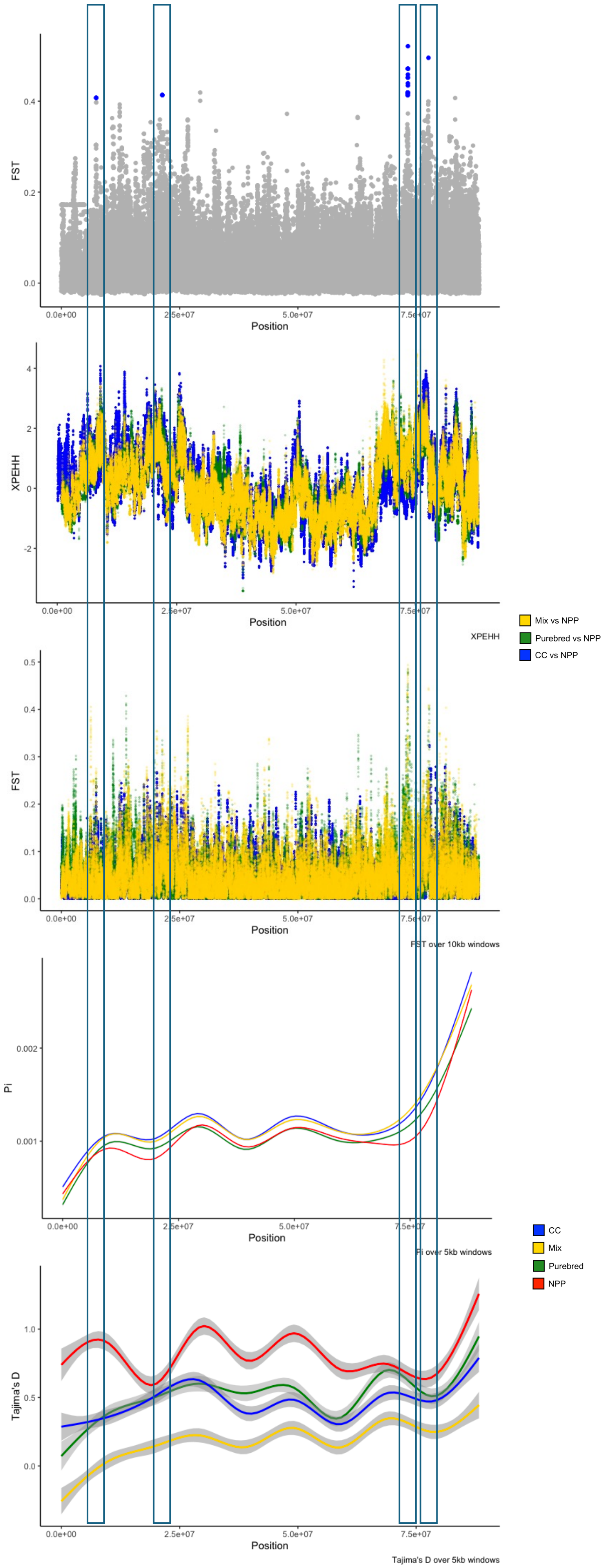

Chromosome 6

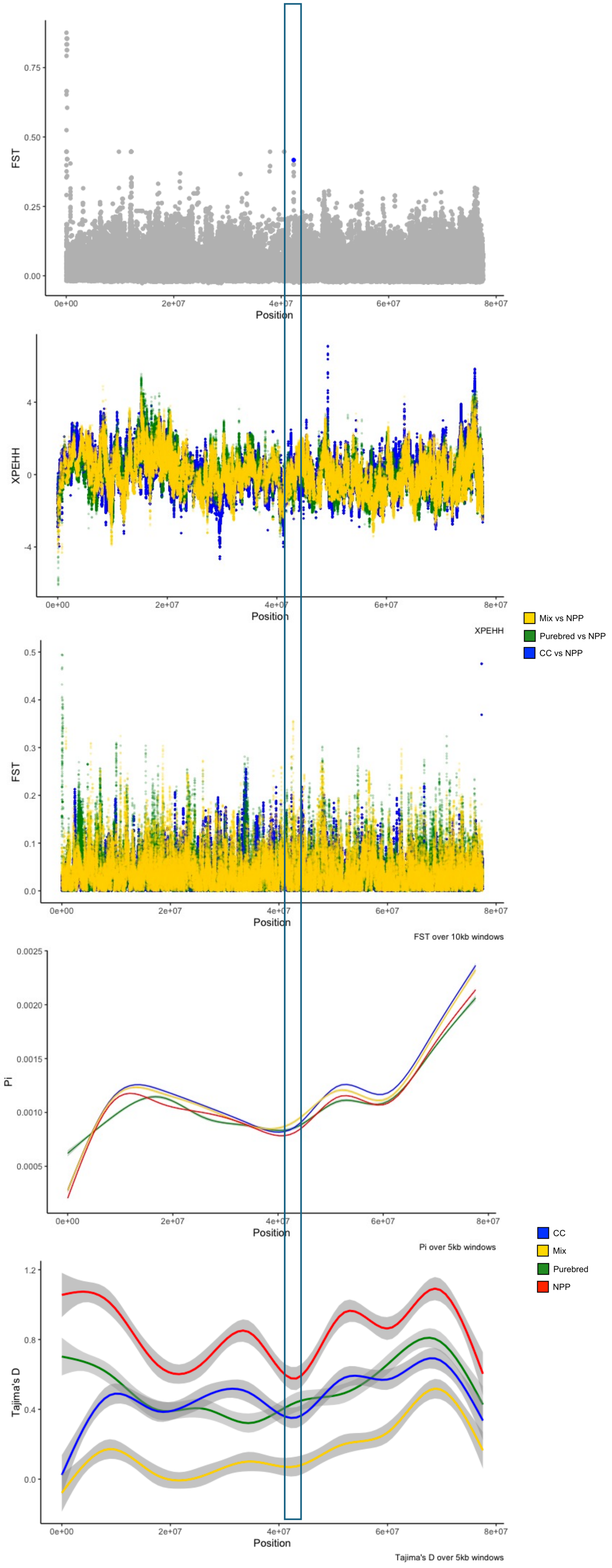

Chromosome 7

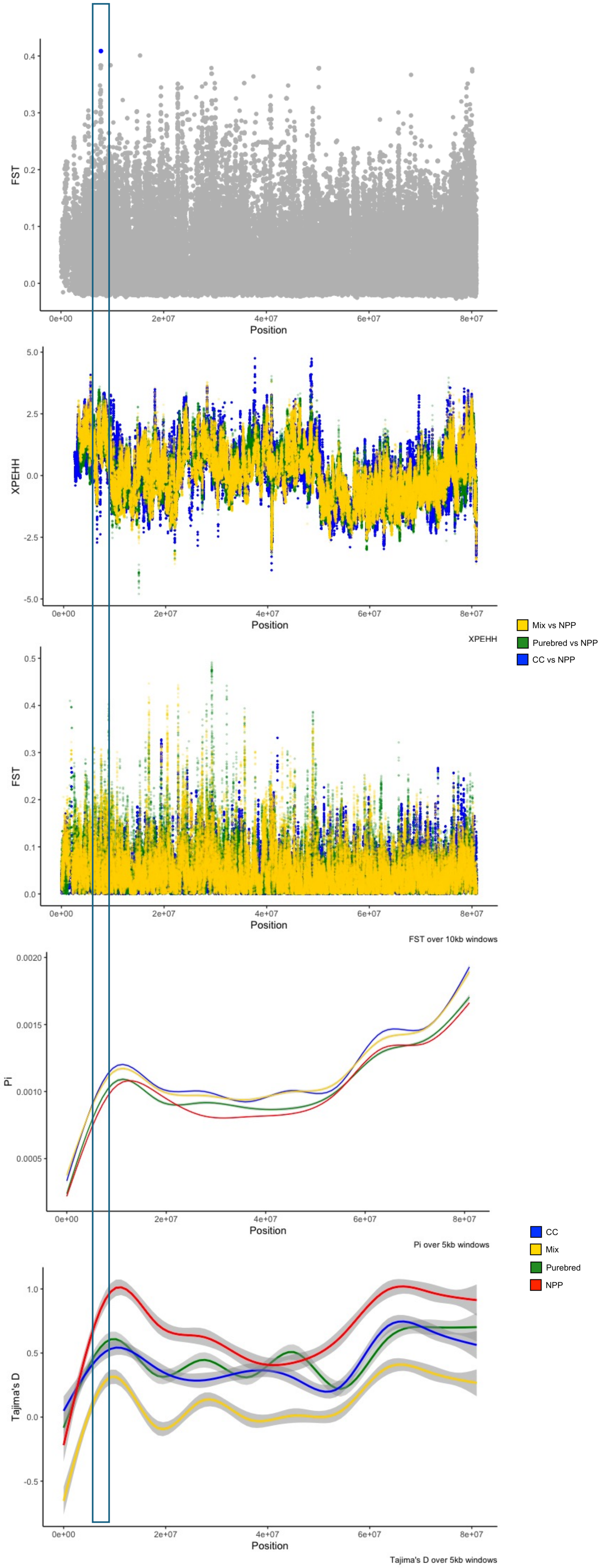

Chromosome 8

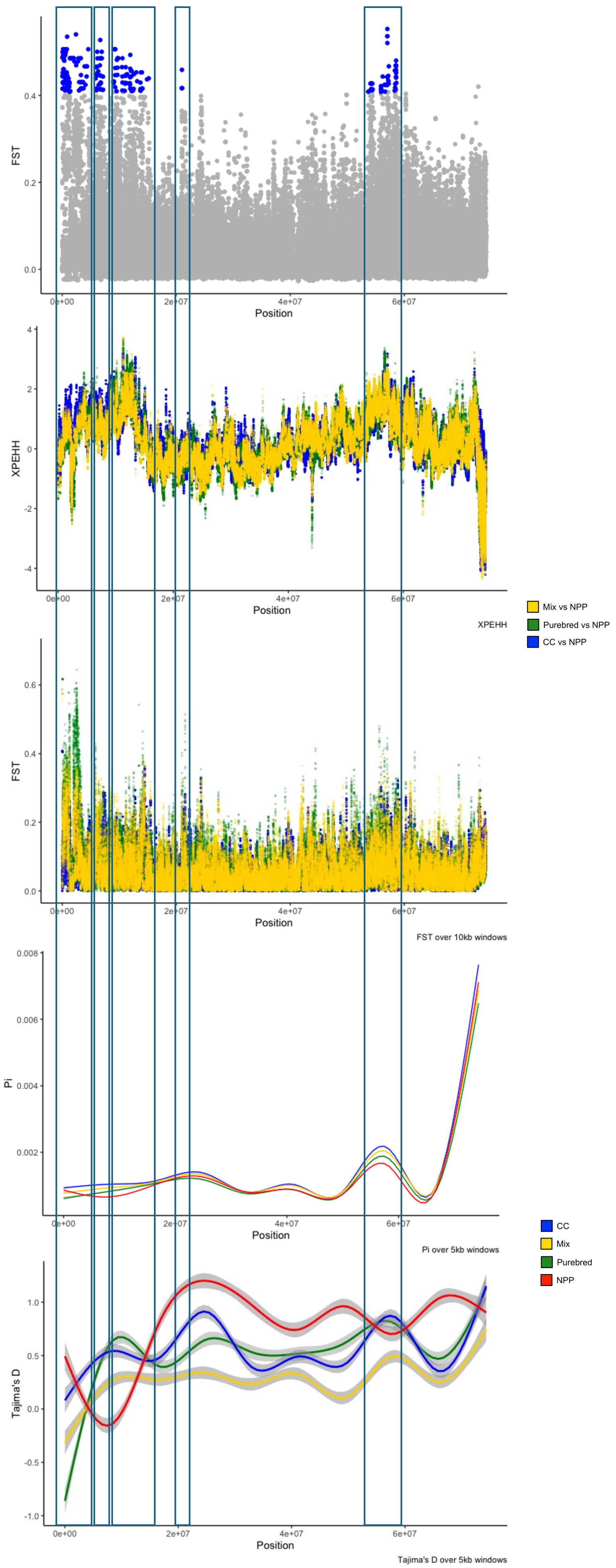

Chromosome 9

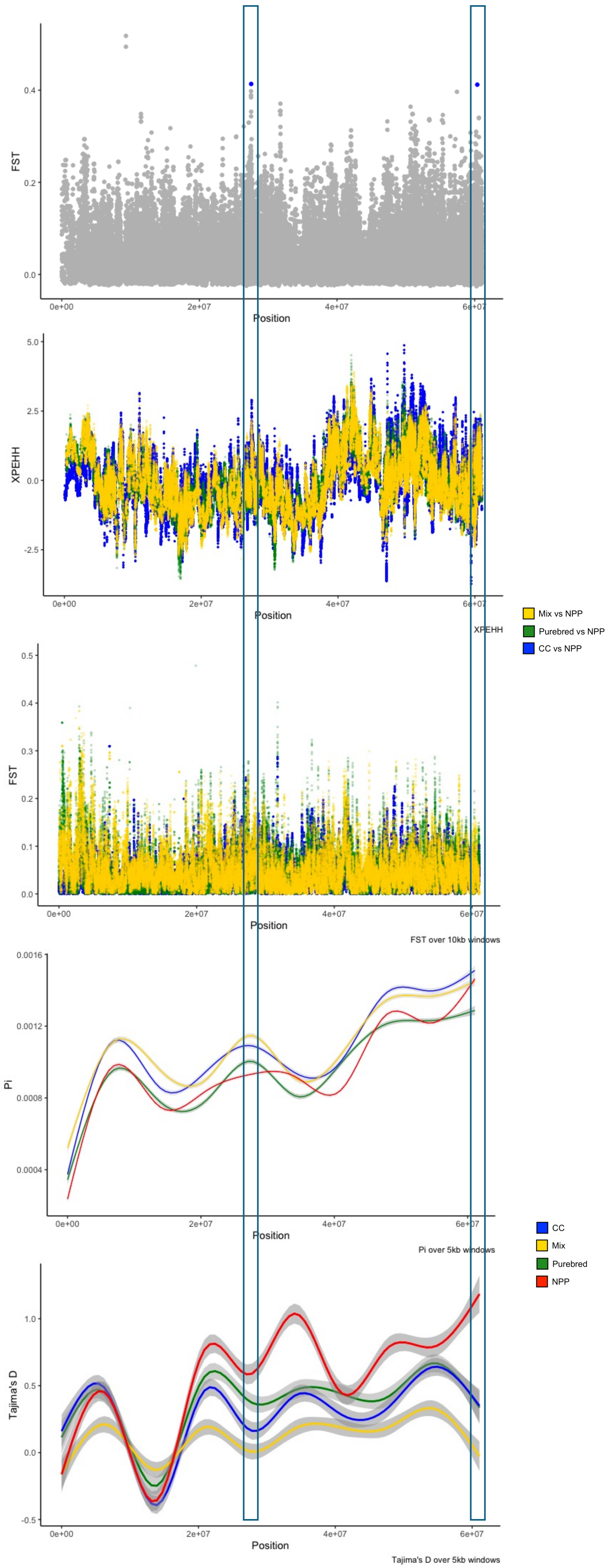

Chromosome 11

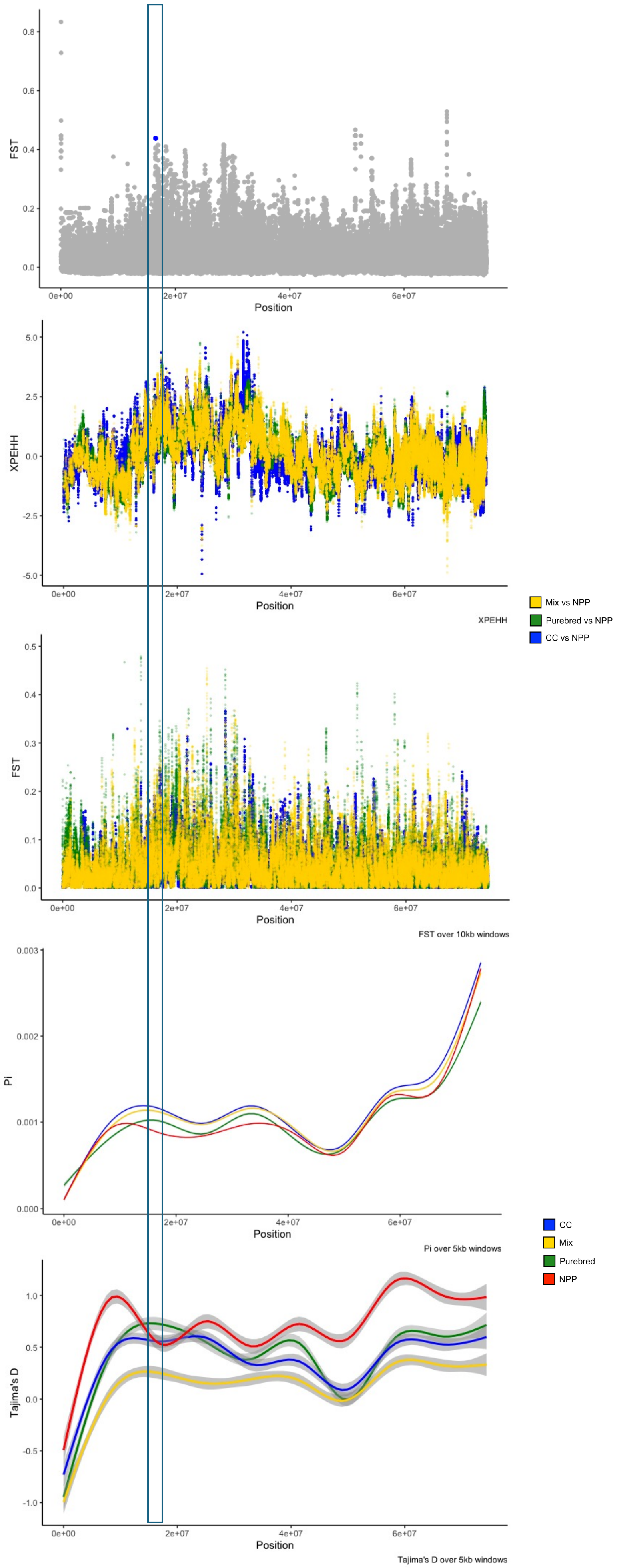

Chromosome 12

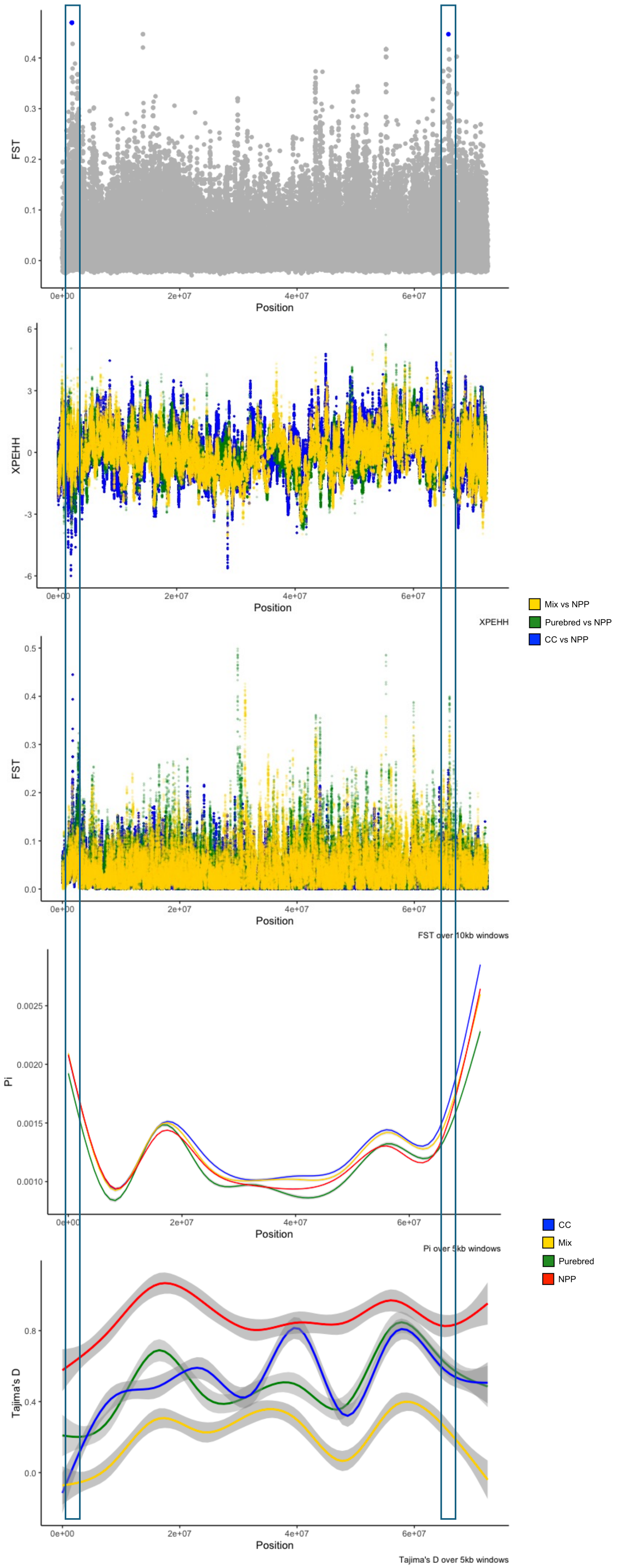

Chromosome 13

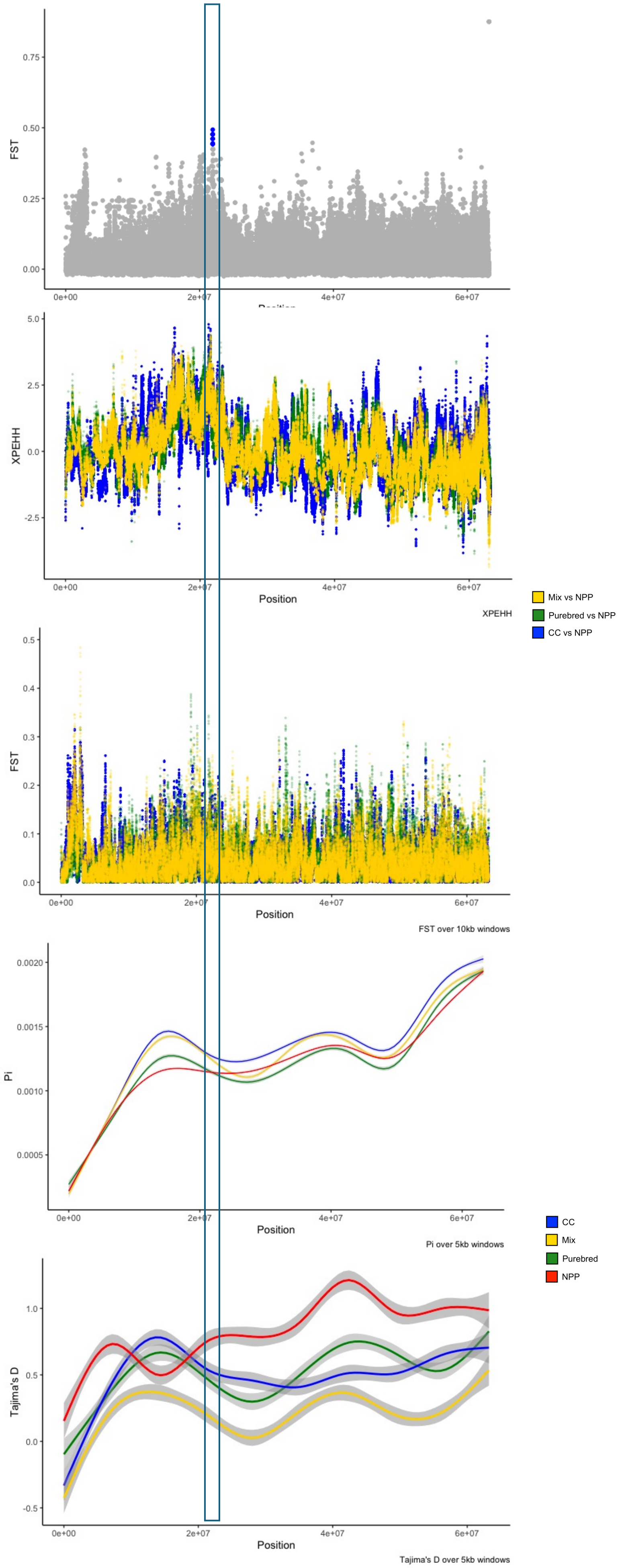

Chromosome 14

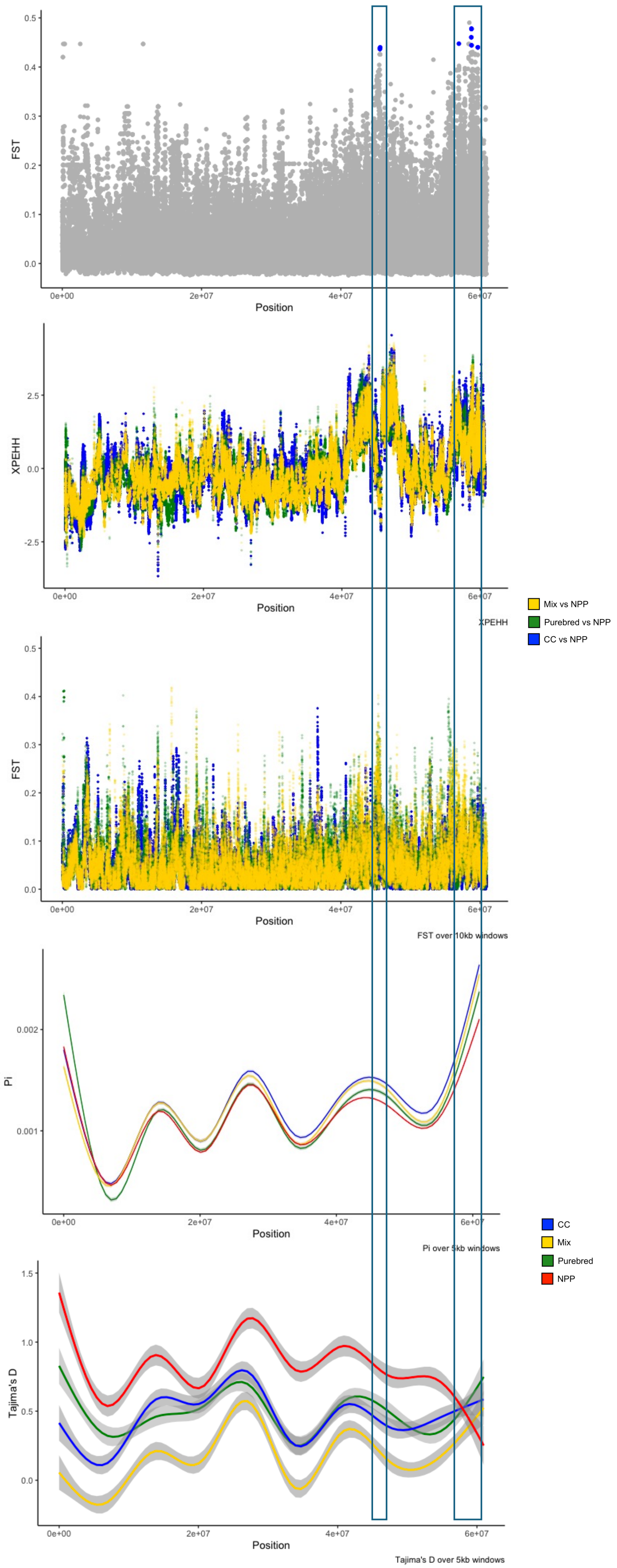

Chromosome 15

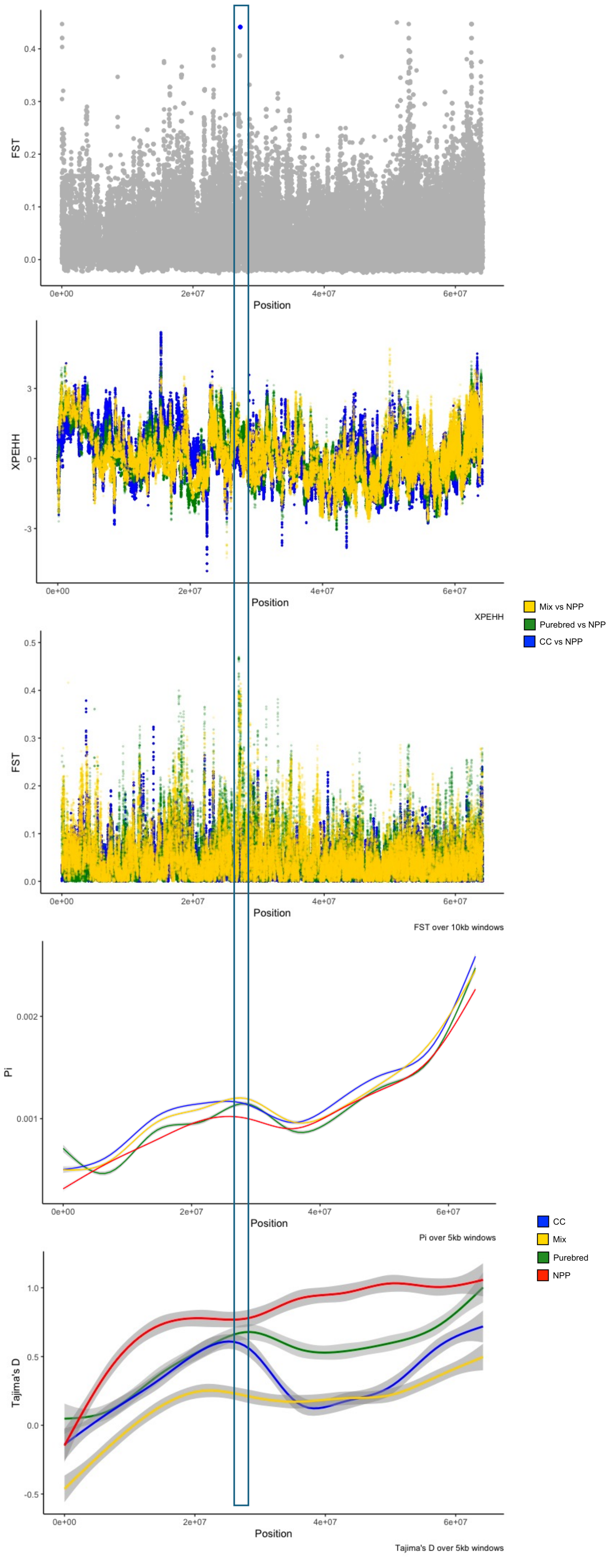

Chromosome 17

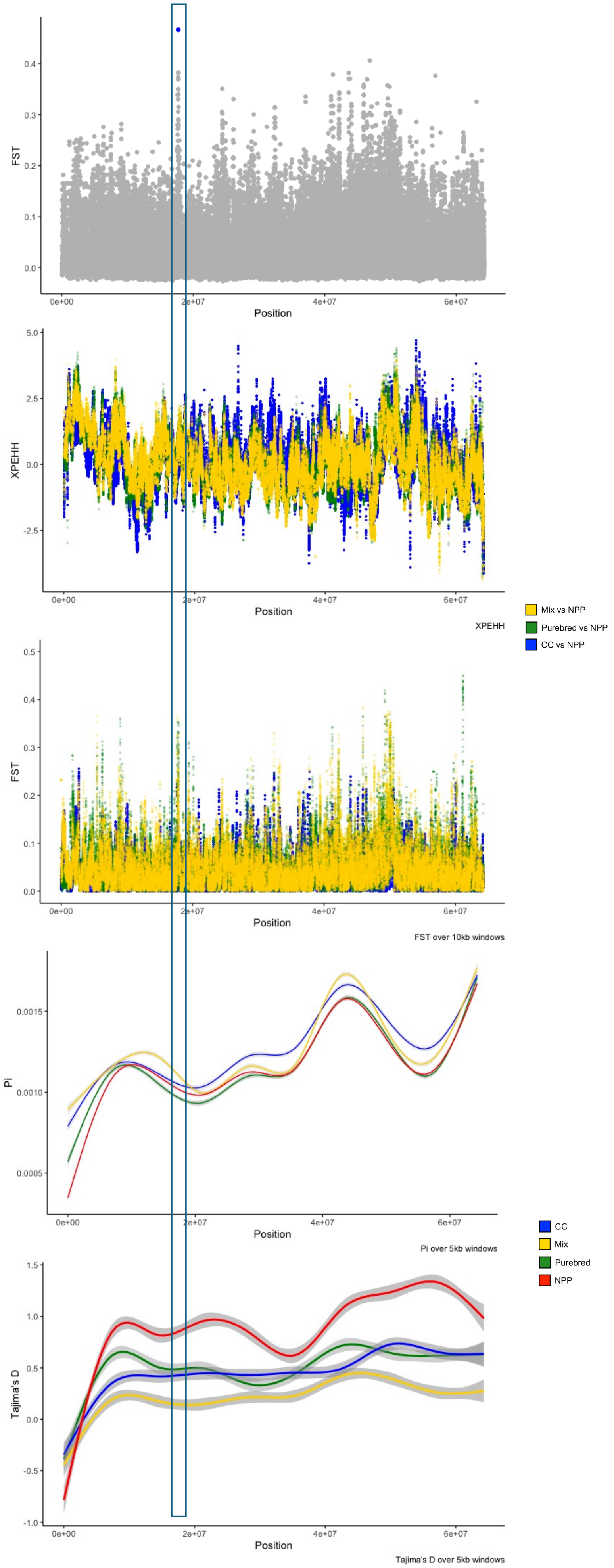

Chromosome 21

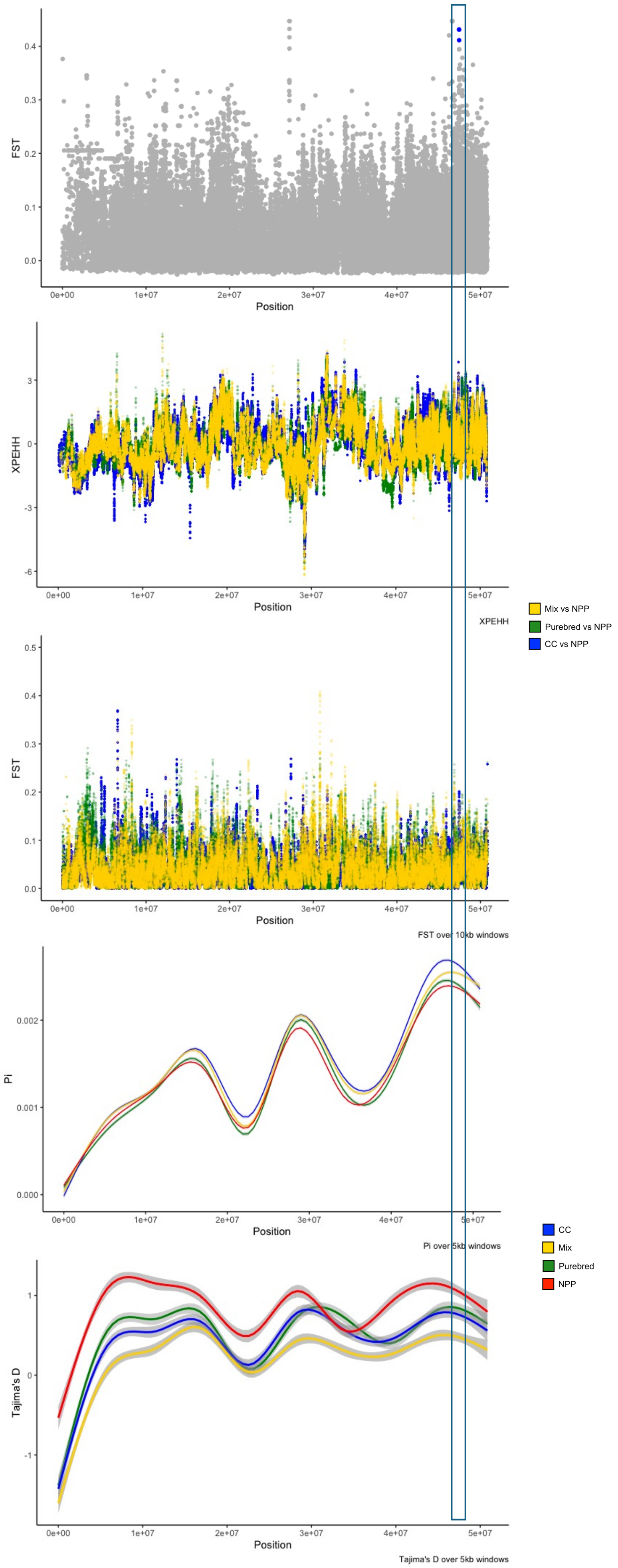

Chromosome 23

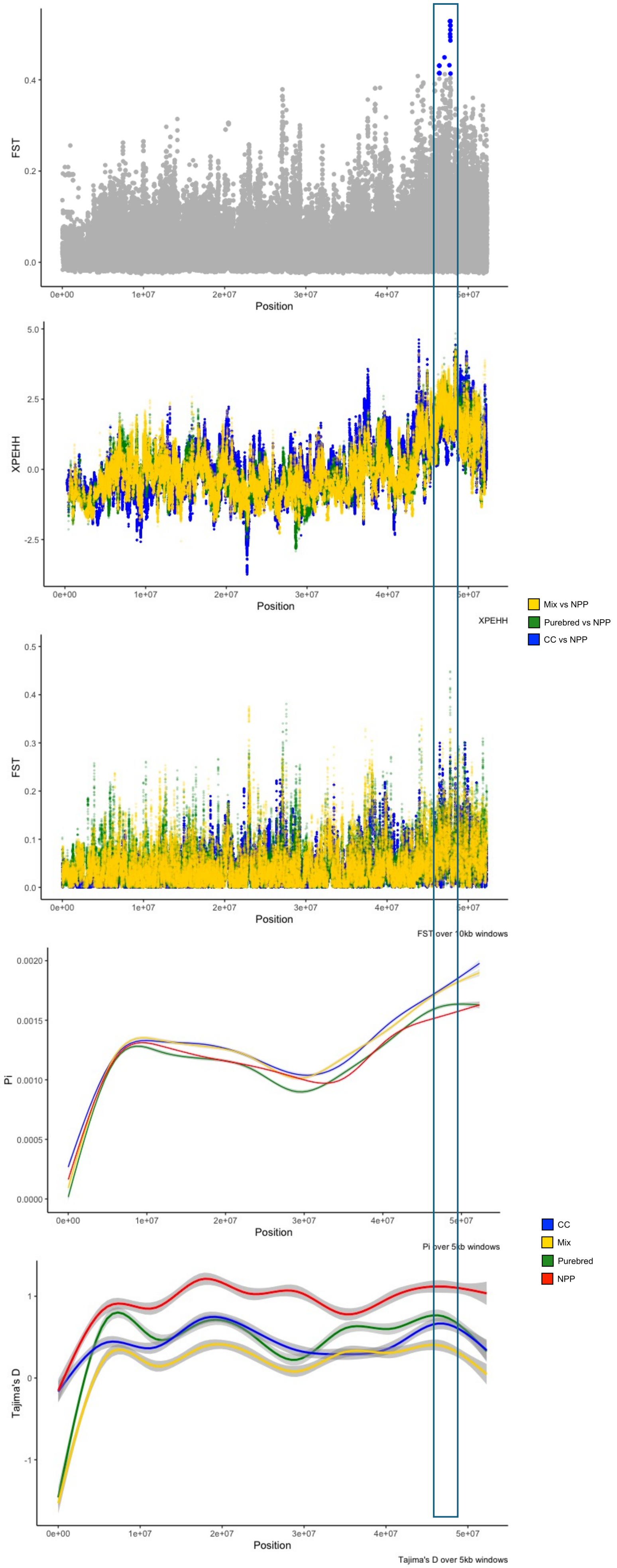

Chromosome 25

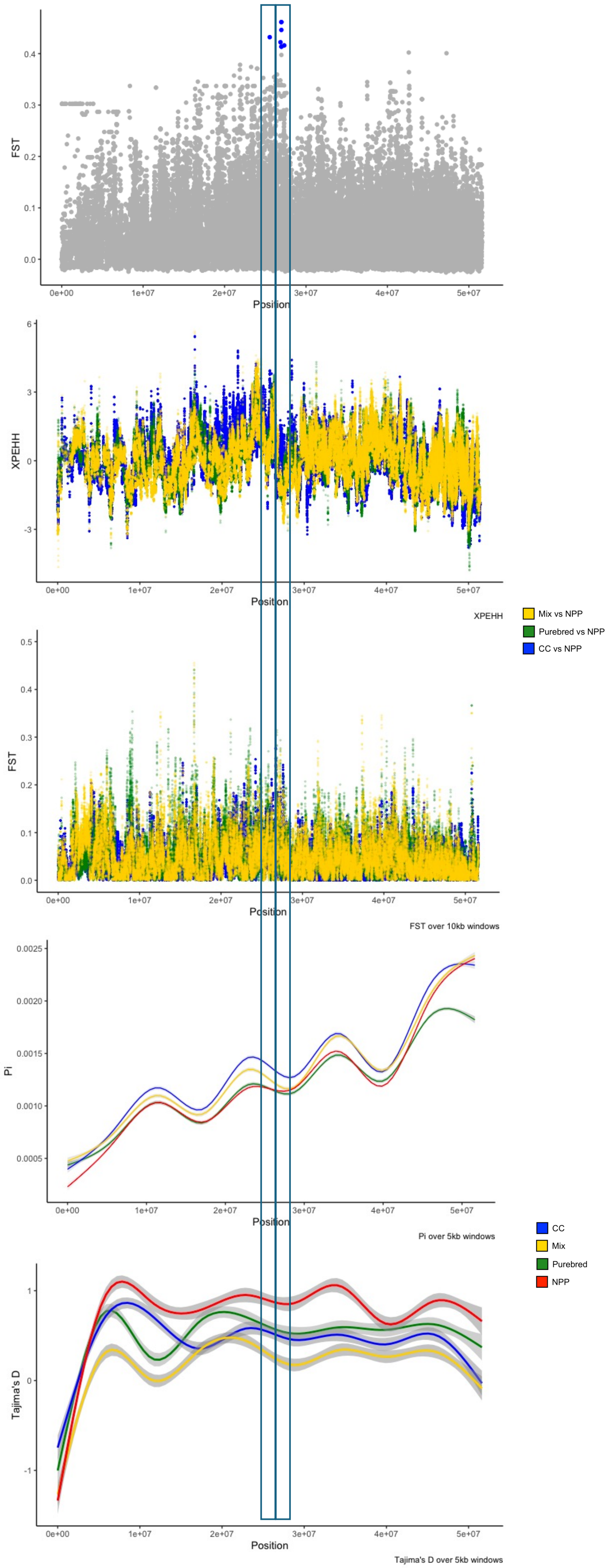

Chromosome 26

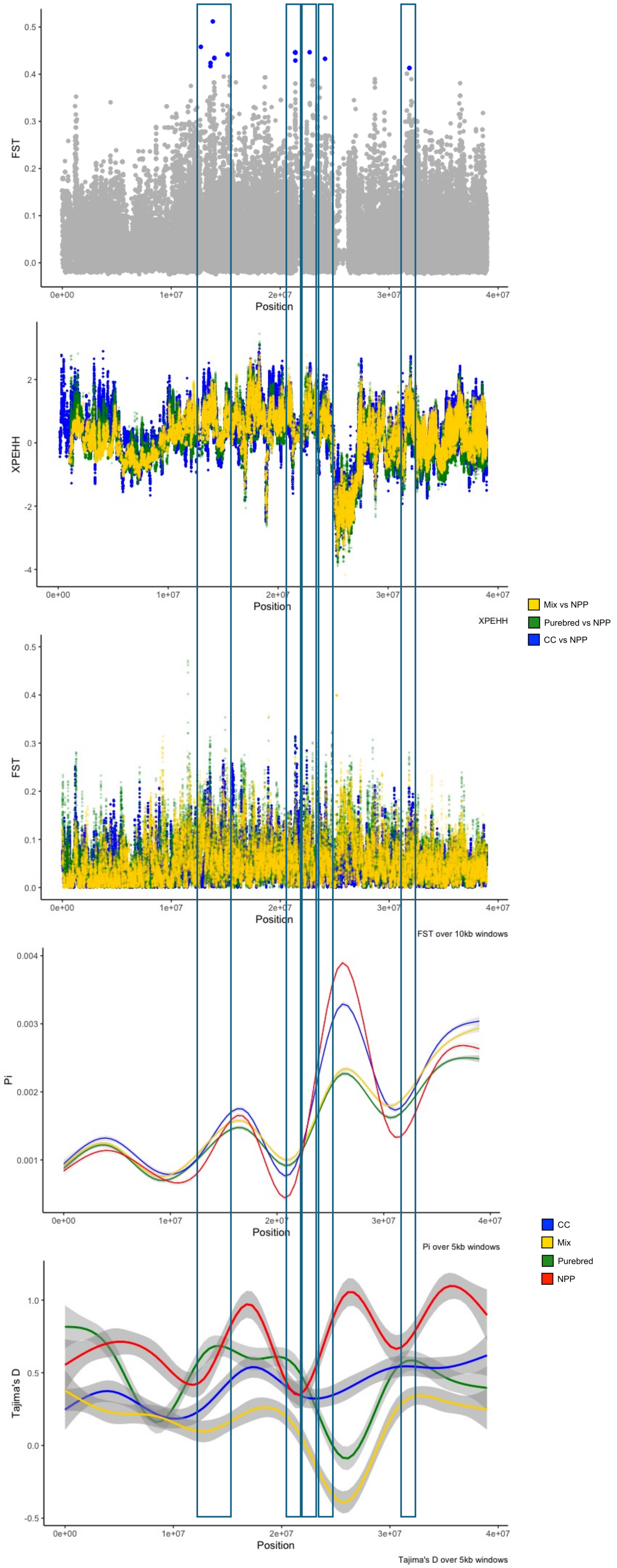

Chromosome 27

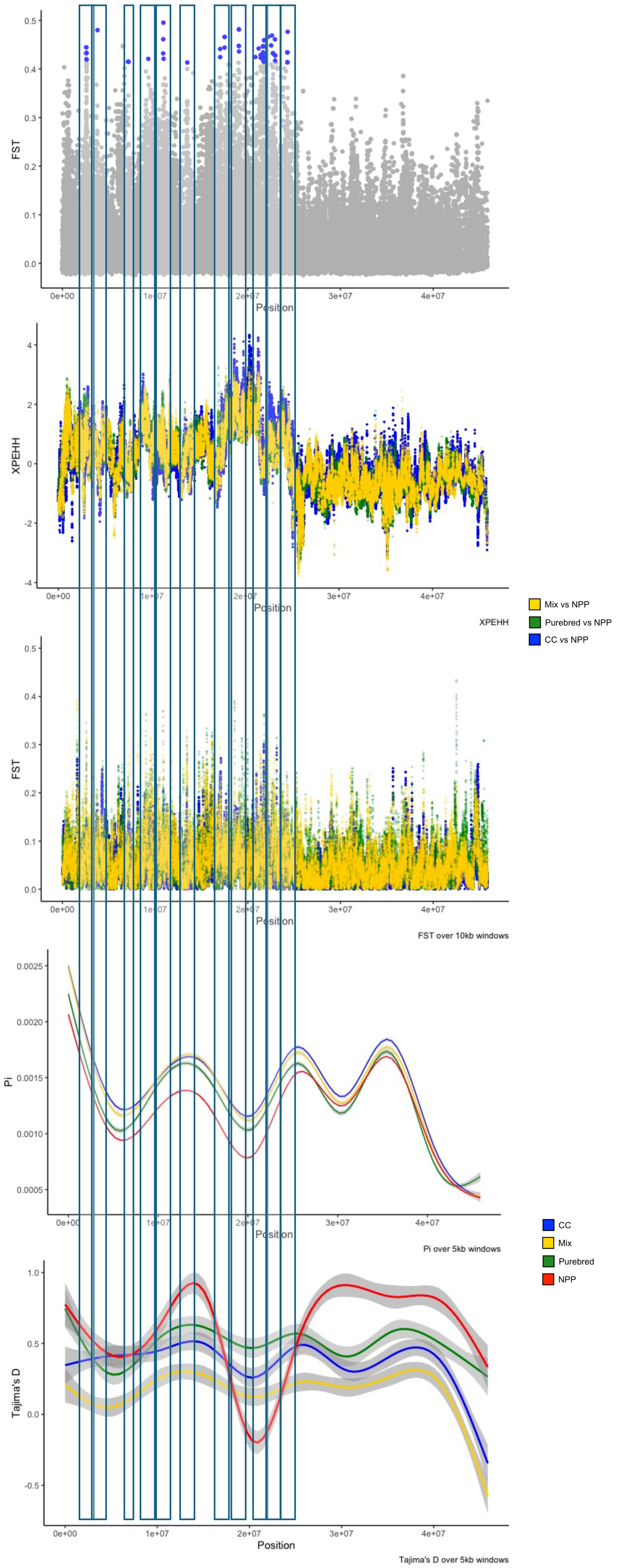

Chromosome 28

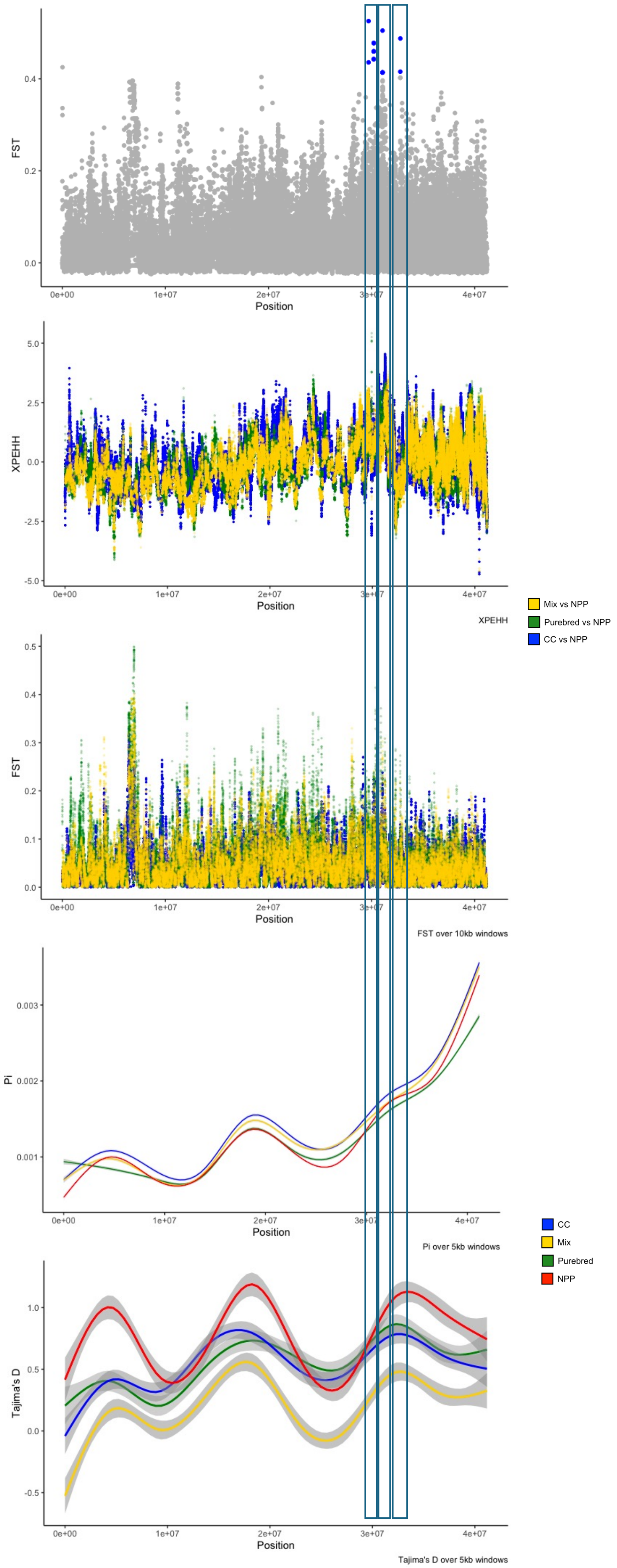

Chromosome 30

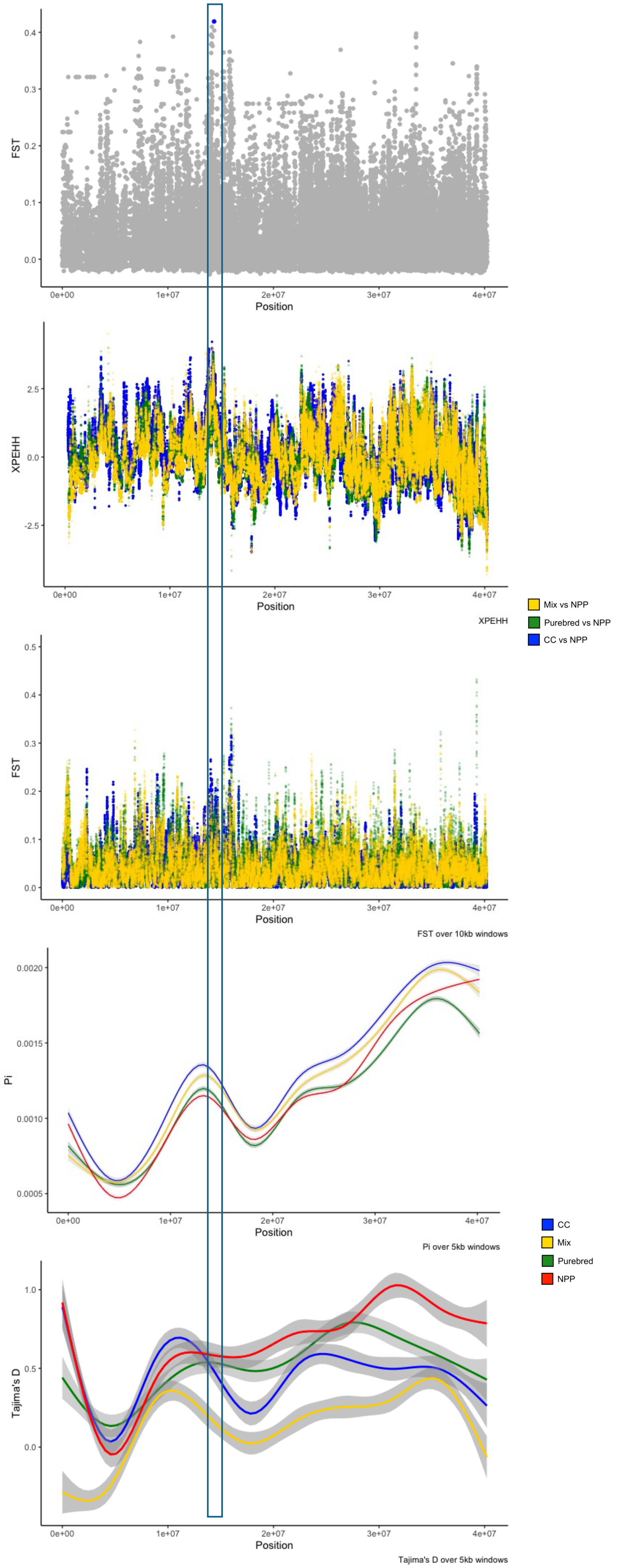

Chromosome 31

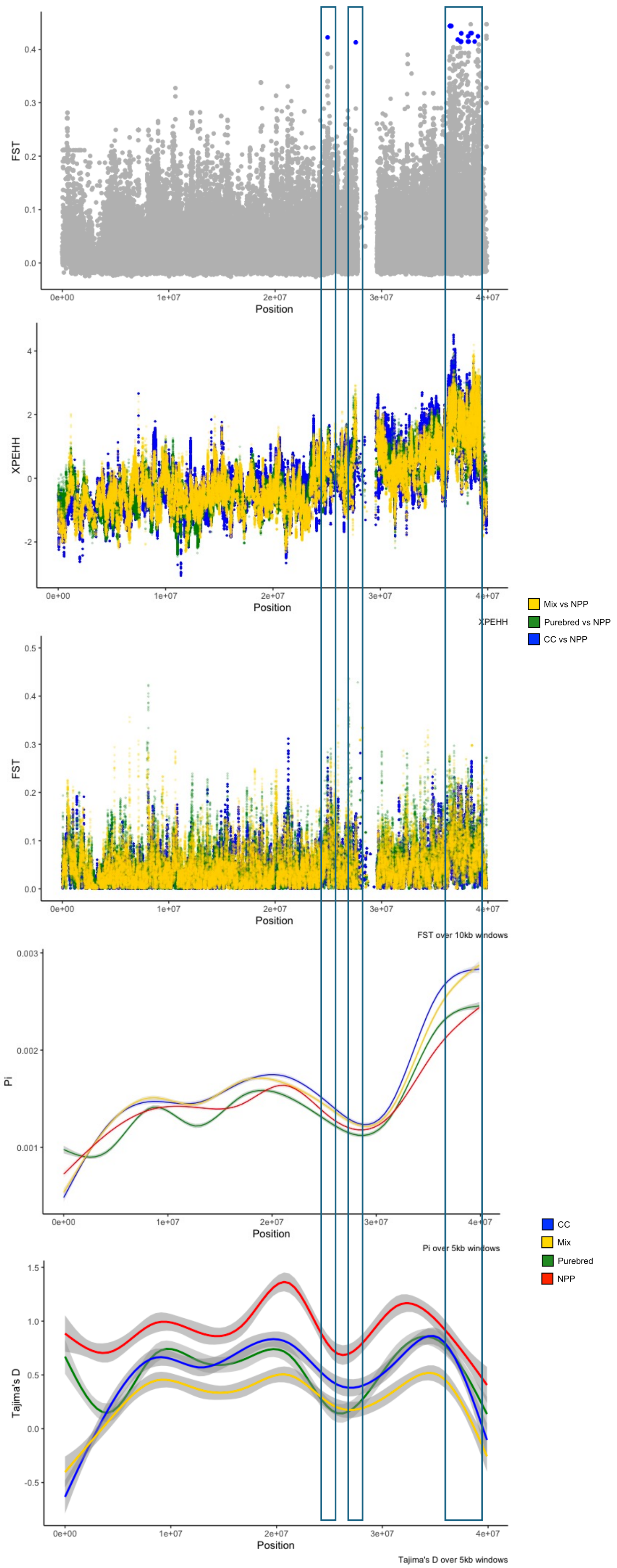

Chromosome 32

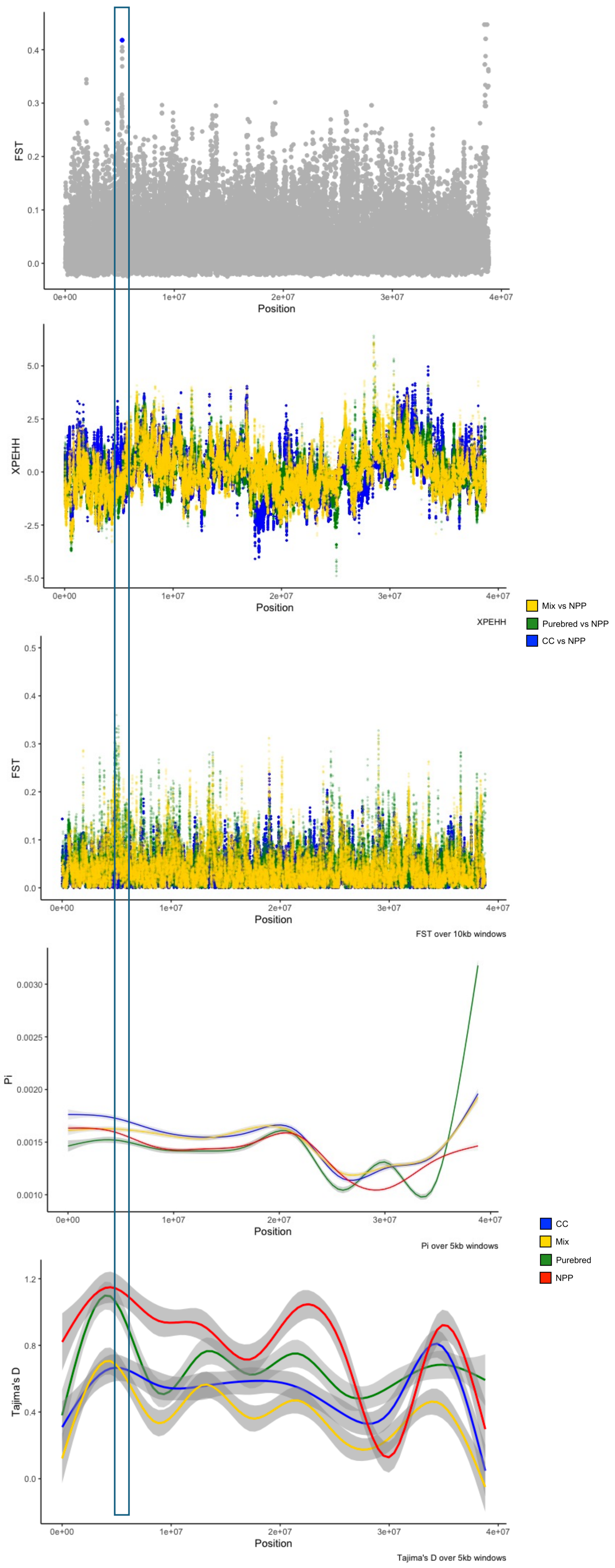

Chromosome 33

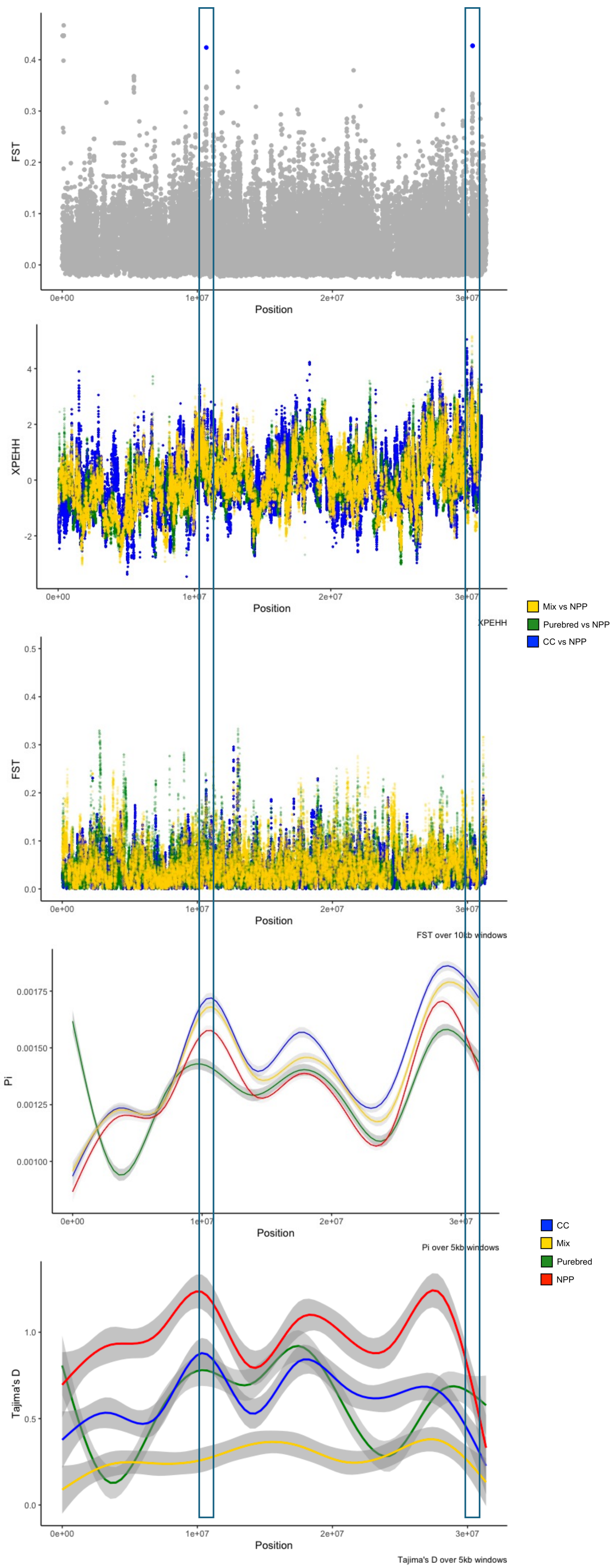

Chromosome 34

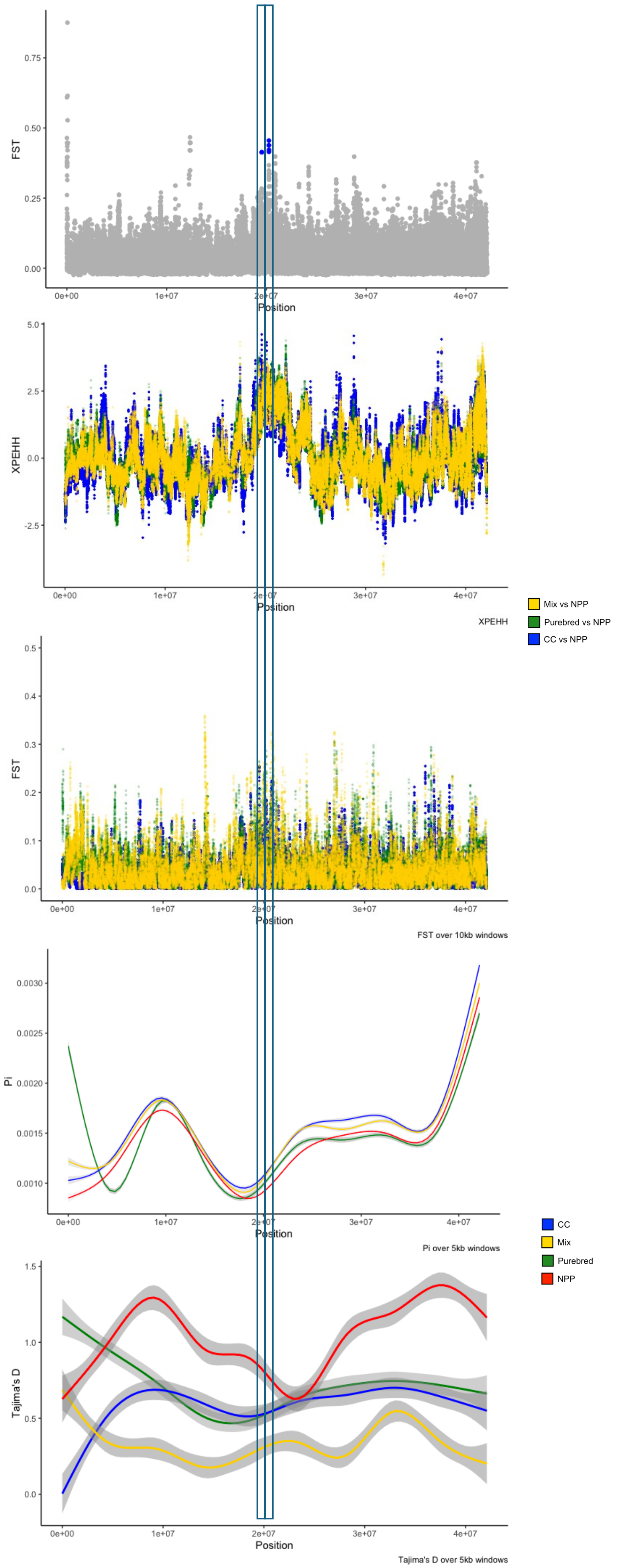

Chromosome 35

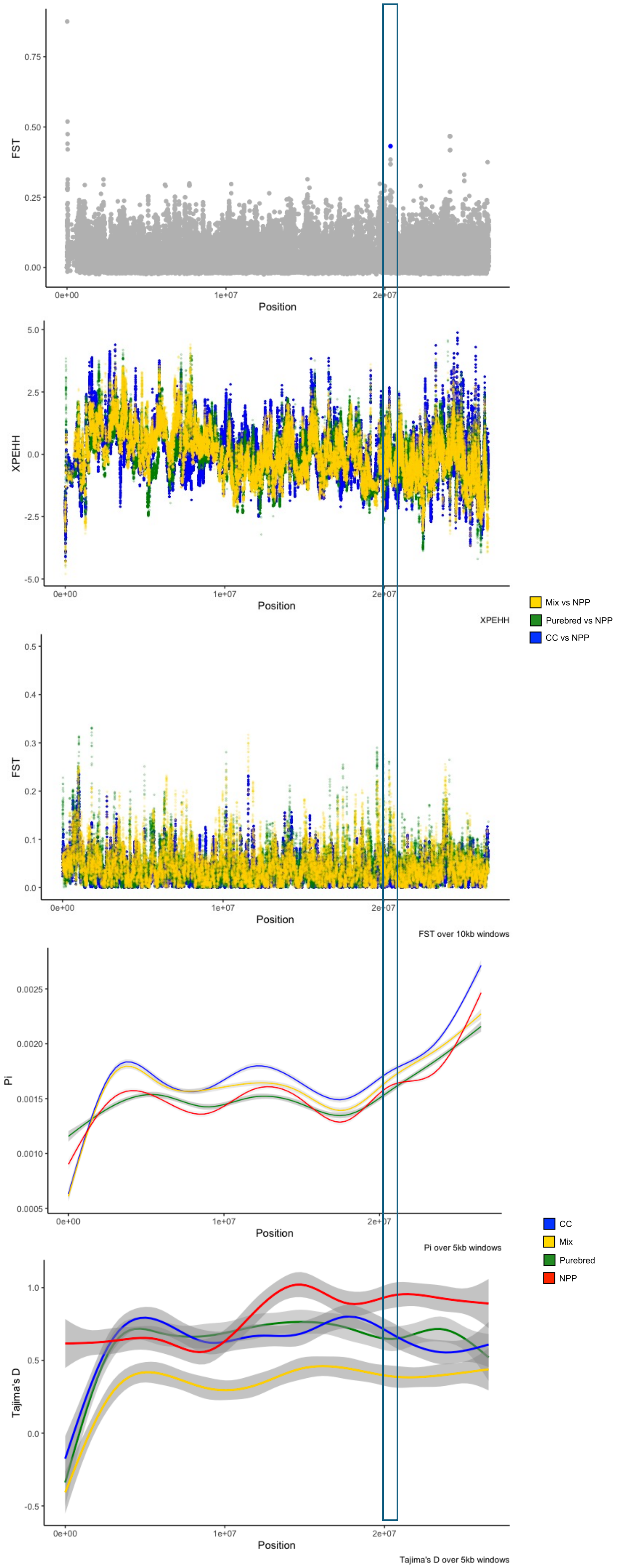

Chromosome 36

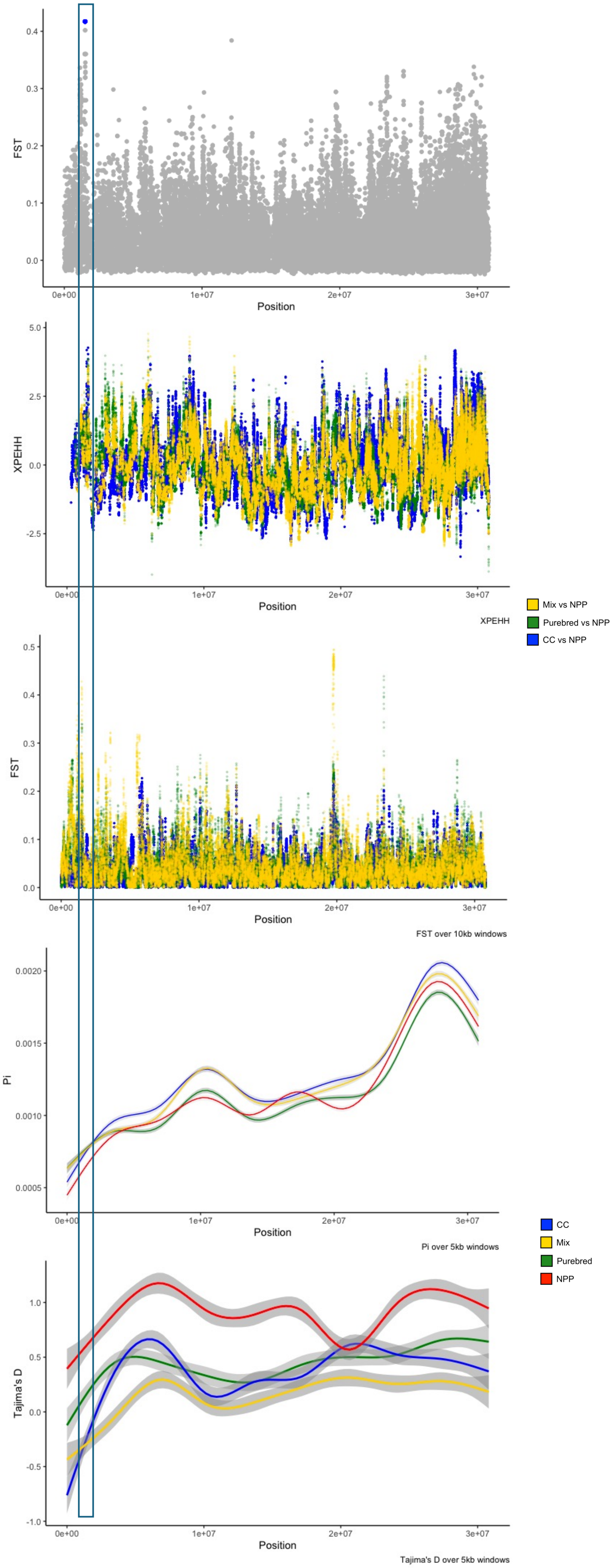

Chromosome 37

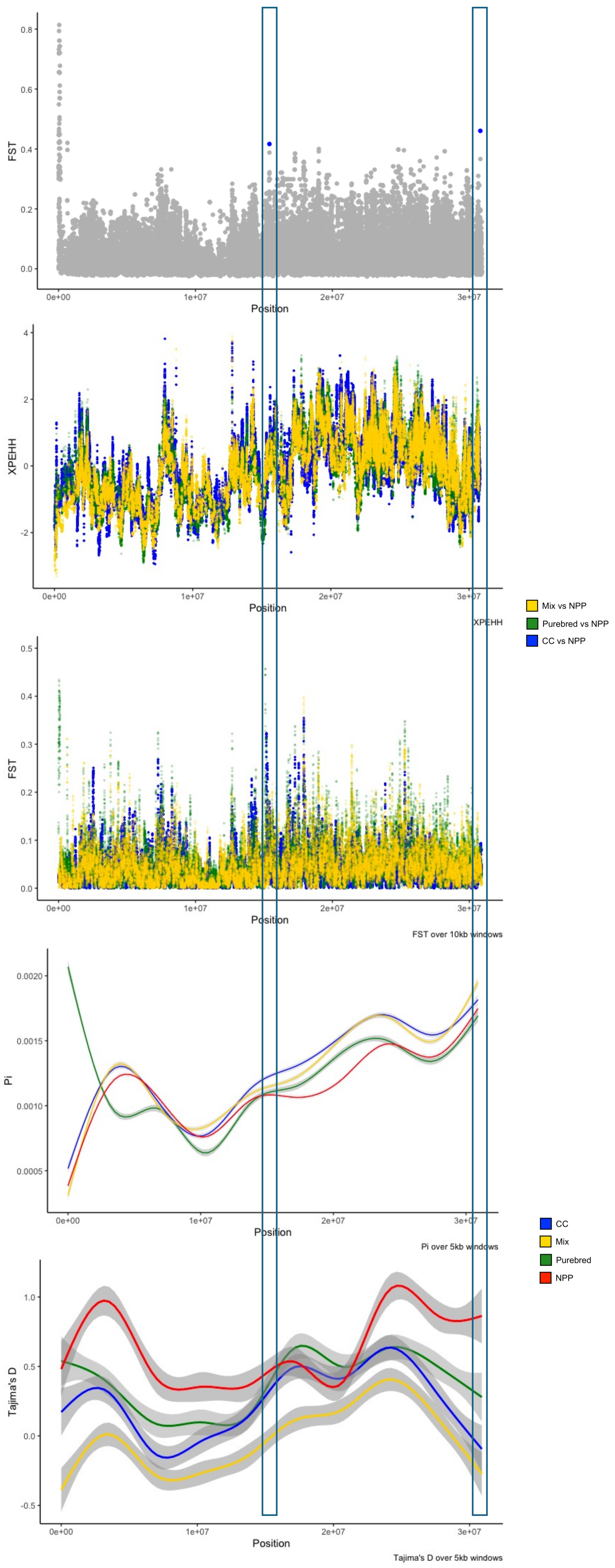

Chromosome 38

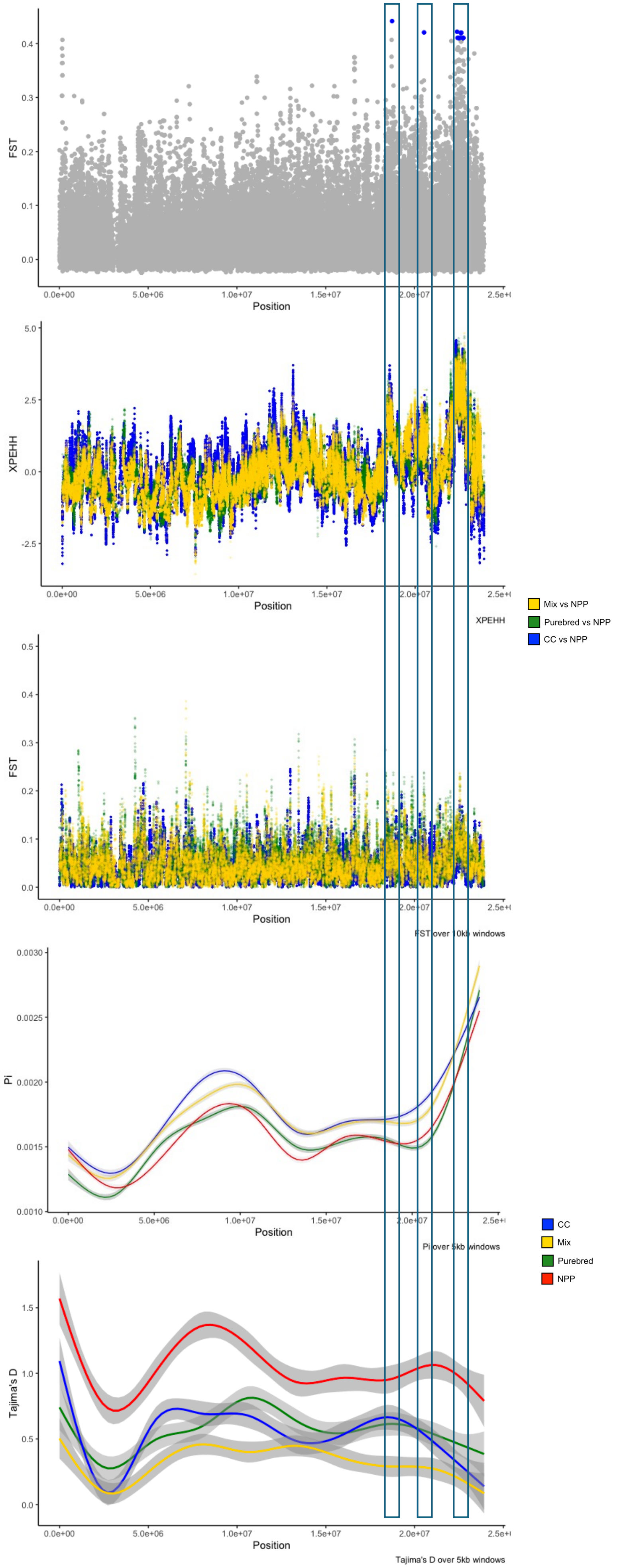

Figure S4. Top associated diseases summary from the Ingenuity Pathway Analysis. The gene dataset that served as the input for IPA includes genes located within 50 kb of the identified genomic regions identified through the OutFLANK outlier analysis. The size of each block is proportional to the number of genes, and the intensity of the coloration is indicative of the -log(p-value).

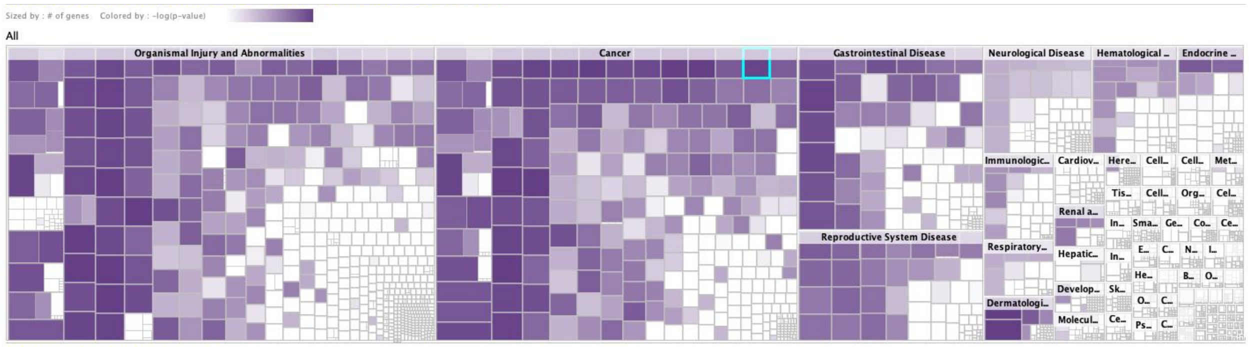

© 2000-2024 QIAGEN. All rights reserved.
